# A Comprehensive Review on Energy Harvesting Integration in IoT Systems from MAC Layer Perspective: Challenges and Opportunities

**DOI:** 10.3390/s21093097

**Published:** 2021-04-29

**Authors:** Golshan Famitafreshi, M. Shahwaiz Afaqui, Joan Melià-Seguí

**Affiliations:** 1 Internet Interdisciplinary Institute (IN3), Universitat Oberta de Catalunya (UOC), Av. del Canal Olimpic s/n, 08860 Barcelona, Spain; mafaqui@uoc.edu (M.S.A.); melia@uoc.edu (J.M.-S.); 2 Faculty of Computer Science, Multimedia and Telecommunication, Universitat Oberta de Catalunya (UOC), Rambla del Poblenou 156, 08018 Barcelona, Spain

**Keywords:** Internet of Things, wireless communication technologies, MAC layer operations, energy harvesting MAC protocols, energy models, energy neutral operation

## Abstract

The Internet of Things (IoT) is revolutionizing technology in a wide variety of areas, from smart healthcare to smart transportation. Due to the increasing trend in the number of IoT devices and their different levels of energy requirements, one of the significant concerns in IoT implementations is powering up the IoT devices with conventional limited lifetime batteries. One efficient solution to prolong the lifespan of these implementations is to integrate energy harvesting technologies into IoT systems. However, due to the characteristics of the energy harvesting technologies and the different energy requirements of the IoT systems, this integration is a challenging issue. Since Medium Access Control (MAC) layer operations are the most energy-consuming processes in wireless communications, they have undergone different modifications and enhancements in the literature to address this issue. Despite the essential role of the MAC layer to efficiently optimize the energy consumption in IoT systems, there is a gap in the literature to systematically understand the possible MAC layer improvements allowing energy harvesting integration. In this survey paper, we provide a unified framework for different wireless technologies to measure their energy consumption from a MAC operation-based perspective, returning the essential information to select the suitable energy harvesters for different communication technologies within IoT systems. Our analyses show that only 23% of the presented protocols in the literature fulfill Energy Neutral Operation (ENO) condition. Moreover, 48% of them are based on the hybrid approaches, which shows its capability to be adapted to energy harvesting. We expect this survey paper to lead researchers in academia and industry to understand the current state-of-the-art of energy harvesting MAC protocols for IoT and improve the early adoption of these protocols in IoT systems.

## 1. Introduction

The Internet of Things (IoT) enables the connection and data transferring over the Internet for a massive number of physical objects, which are equipped with distinct hardware and software to enhance a wide range of applications and services [1]. These enhancements as part of the IoT paradigm aim at adding value to every aspect of human life and society, from digital hospitals in healthcare services to process management in industrial automation [2]. According to the Cisco Annual Internet Report [3], it is expected that the number of connected devices will increase from 18.4 billion in 2018 to 29.3 billion devices by 2023. Hence, providing sufficient energy to maintain this massive number of connected devices will be a challenging. The analysis from “The Shift Project” [4] conveys that the increasing trend of IoT connected devices leads to a Computational Annual Growth Rate of 4.5% in the expected energy consumption of IoT deployments (from 2312 TWh in 2015 to 4350 TWh in 2025). According to these predictions, in the near future, powering up IoT devices with conventional batteries with a limited lifetime, which requires frequent replacement, is a concerning issue and may cause system failure [5]. Moreover, since IoT systems have spread across many different use cases, from healthcare and industrial to transportation and residential, end devices may be located in hard to reach and hazardous areas, where the maintenance and frequent conventional batteries replacement make the usage of them inefficient and costly [6]. This means each year, billions of batteries are accumulated in landfills, which negatively impact the environment, such as ecotoxicity and water pollution.

The limited lifetime of the conventional batteries, which increases the maintenance cost, number of replacements, and negative impact on the environment, in a system with a few devices do not raise an issue, whereas, in networks with millions or even billions of devices, it becomes a significant issue. Since these battery limitations threaten the rapid development of the IoT paradigm, academia and industry have become interested in extending the lifetime of IoT devices while maintaining optimal performance. For this purpose, power management techniques, including energy-efficient methods (e.g., light-weight protocols, scheduling optimization, and low power transceivers) or energy harvesting techniques (e.g., ambient energy harvesting, and dedicated energy harvesting) [7], and energy conservation methods in IoT devices are currently hot topics. Alongside the energy-efficient techniques, which reduce the networks’ energy consumption, recent innovations in IoT technologies such as portable devices with small batteries lead to introducing energy harvesting technologies as a promising solution to provide enough energy for them [8] and prolong the lifetime of the network. The authors in [9] emphasized the fundamental role of energy harvesting technologies in IoT systems by imparting that the increasing interest of academia and industry in energy harvesting technologies leads to a growth in the energy harvesting global market from 360.6 million dollars in 2020 to 987.09 million dollars by 2028.

**Figure 1:**
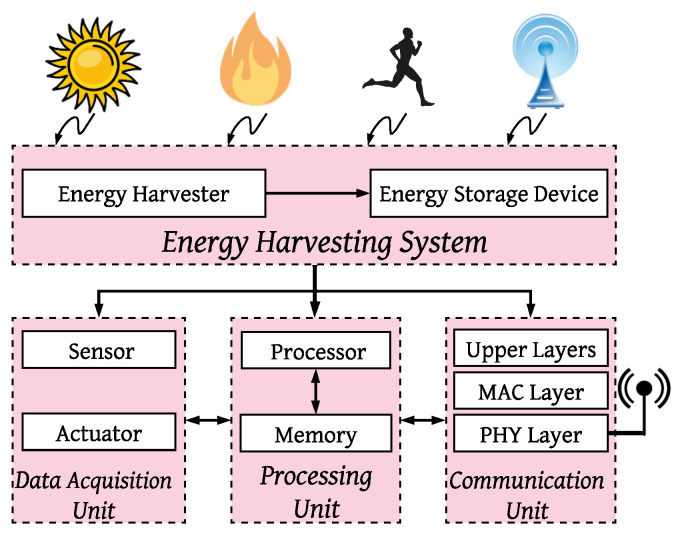
General schematic of an IoT node equipped with energy harvesting.

Although energy harvesting technologies provide more energy to IoT systems, to satisfy their possibility of integration with IoT systems, some parameters such as size, type of the end-user device, and IoT application need to be taken into account. To understand how energy harvesting technologies are envisioned to be supported in IoT, different authors in the literature [10,11] provide the schematic in Figure 1. This figure is based on these papers, which lists the key components required to support energy harvesting at the IoT system’s sensor level. The top layer is responsible for harvesting energy. Three parts of the bottom layer make wireless communication possible, manage the entire device, and include sensors and actuators (from right to left). In systems like Figure 1, the Energy Neutral Operation (ENO) condition is achieved if the energy harvester provides energy greater than or equal to the required energy of the system. However, fulfilling this condition under specific considerations (e.g., small size of the energy harvester) and the whole system’s requirements remains a gap in the literature.

### 1.1. Motivation

The increasing trend in the number of connected devices in the IoT paradigm suggests that powering up these devices with conventional batteries requires frequent battery replacement, which is not efficient and leads to environmental contamination. Hence, there is a need to improve the efficiency of IoT technologies to be able to prolong the lifetime of these systems. However, due to the different characteristics of each IoT technology, achieving this sustainability improvement is a challenge.

**Figure 2:**
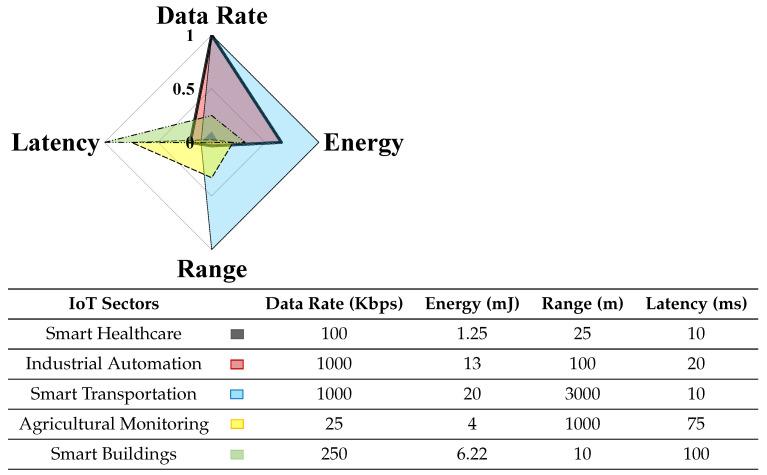
Different KPIs for IoT use cases [12,13,14,15,16,17,18].

The IoT market is providing different wireless communication technologies to support the various Key Performance Indicators (KPIs) of the IoT applications at the communication level (cf. Figure 2). Depending on the implemented wireless communication technology, each IoT system will have different performance requirements, where energy consumption is one of the most critical aspects. Hence, there is the need to study energy consumption in different IoT wireless communication technologies to understand its impact on IoT performance. Since there is no proper characterization of these technologies in terms of energy requirements in the literature, there is a need for a unified energy model approach. After understanding the energy requirements at the IoT communication level, different solutions to improve the sustainability of the IoT systems towards the ENO property can be introduced. Within the available solutions, energy harvesting is a popular one for improving sustainability in IoT. However, based on the current literature, the energy provided by energy harvesting is not always sufficient with the IoT communication technologies because of the limited and intermittent behavior of energy harvesting energy sources. Hence, the challenge is to integrate energy harvesting in wireless communication technologies without impacting the system’s performance.

For successful integration of energy harvesting within IoT systems, there is the need to optimize the energy consumption in wireless communication technologies at different IoT layers. For instance, an adaptation to the channel condition or energy-aware routing protocols can optimize the energy consumption level at the physical and network layer, respectively. Nevertheless, since the Medium Access Control (MAC) layer is responsible for scheduling the data frame transmissions and faces fundamental communication challenges (e.g., collision frames, the overhead of control packets, idle listening, unused idle slots, synchronization, and others), it consumes most of the energy budget of wireless communications. Thus, there is the need to adapt this layer to make the IoT systems sustainable and compatible with energy harvesters while keeping these modifications compatible with existing wireless technologies.

### 1.2. Contribution

As highlighted in the introduction of this survey, the MAC layer in wireless communication has an essential role in optimizing the IoT systems’ energy usage. To understand the characteristics of different MAC mechanisms and their limitations in the IoT scenario, a MAC categorization is necessary. Since there are diverse wireless technologies already being deployed at the communication level of the IoT systems, to understand the requirements of each technology in terms of energy, given that MAC layer operations are power-hungry, the analysis of energy consumption needs to be performed based on these operations. The study of energy consumption for communication technologies is achieved by energy consumption models, which can be obtained based on real hardware measurements, simulations, or analytical models. Before explaining the already existing energy harvesting-based MAC modifications and enhancements in the wireless communication technologies literature, together with their benefits, drawbacks, and compatibility between energy harvesters and its application to the different IoT use cases, it is necessary to estimate the amount of energy that each energy harvester provides to the system. Thus, the characteristics of the energy harvesters and their energy sources need to be studied. We expect the provided information lead the researchers in academia and industry to understand the limitations of the existing works and promote a change of thinking for early adoption of energy harvesting techniques within the IoT paradigm.

In this survey paper, we searched for the most relevant articles in available databases among the high-quality journals and conferences and their relevant references that were cited papers during the past two decades. We organized our search based on a selected keyword list which includes the most common and relevant keywords to this topic. To the best of our knowledge, the wireless communication technologies of the IoT paradigm have not been compared based on their actual amount of power consumption before in the literature. Also, to the best of our knowledge, the compatibility of IoT communications with available energy harvesters is studied from a MAC layer perspective in a structured manner for the first time in our survey paper. To summarize, this survey paper includes the following contributions:

- We extensively review the already existing energy-aware MAC protocols to develop a categorization that identifies the various dimensions of proposed MAC additions to enable the concurrent use of energy harvesting.
- We comprehensively study the available wireless communication technologies to highlight their compatibility with the IoT paradigm and their existing MAC layer features, accompanied by different MAC optimization techniques for each technology. Our work takes current literature to develop a unified approach to analyze energy models, contributing to a better understanding of energy requirements and shortages of the IoT systems in terms of wireless communication technologies.
- We contribute with an analysis of the functionalities and characteristics of existing energy harvesters and their suitability for the wireless communication technologies in the IoT paradigm.
- We thoroughly study the functionality of existing energy harvesting MAC protocols in the literature, their benefits and drawbacks, to understand the available integration of energy harvesting techniques at the MAC layer and their limitations.
- We comprehensively review the energy consumption of the different levels of IoT systems with a focus on the MAC layer operations. This study specifies the energy wastage through MAC anormalies, which determines the essential considerations to enable energy harvesting techniques at the MAC layer.
- We provide an extensive guideline for open issues and research challenges for energy harvesting MAC protocols within IoT systems.

The remainder of this paper is organized as follows. In Section 2 the state of the art of this paper is highlighted. Section 3 presents the categorization of the energy-aware MAC protocols for IoT systems. Then potential wireless communication technologies and their energy models for IoT systems are explained in Section 4. Section 5 is described the available energy harvester technologies and their applicability with IoT systems. The existing energy harvesting MAC protocols in the literature are categorized based on their mechanisms and then explained in Section 6. Some challenges regarding the MAC layer and open research directions and future works are highlighted in Section 7. In the end, in Section 8 some final remarks are given.

## 2. State of the Art

There are numerous existing MAC protocols in the literature, where each of them has distinct benefits and drawbacks. To meet the requirements of the existing wireless communication technologies, specifically in terms of energy consumption, the MAC layer protocols of these technologies may adopt different mechanisms. However, the defined MAC protocols, for the current wireless communication technologies, do not consider the energy harvesting paradigm in their design procedure. Thus, to support energy harvesting techniques on the specific MAC protocol, it is necessary to understand the implications of these techniques on the benefits and drawbacks of that protocol. The first step to understand these requirements is to review the available energy harvesting MAC protocols in a structured way. In the past decade, several studies have defined various categorizations for the energy harvesting MAC protocols in IoT systems and highlighted the requirements of different MAC mechanisms to support energy harvesting technologies. This section will explain the related works regarding these categorizations.

One of the earliest studies that explored the possibility of enabling energy harvesting techniques in the MAC layer of IoT systems by reviewing previous works was proposed in [19]. In this work, the authors explain the role of energy harvesters in IoT systems and the requirements of these networks. The proposed energy harvesting MAC classification was built based on the adopted optimization techniques in designing the protocols, such as load balancing, contention reduction, or wake-up time awareness. In this work, the authors study the approaches of the MAC protocols which have enabled the available ambient energy harvesting methods, and then they highlight the strengths and drawbacks of each energy harvesting MAC protocol. Although the authors provide detailed information about the functionality of the selected protocols, the selection of state-of-the-art requires an update to include all the existing energy harvesting MAC protocols in the literature. Also, the challenging issues in designing an energy harvesting MAC protocol and the possible future guidelines to improve the existing energy harvesting-based MAC protocols were not considered. Similar to the previous literature review paper [20], briefly explains the primary sources of ambient energy for energy harvesting IoT systems and the architecture of the energy harvester node. In this work, the author emphasizes the ENO condition as the main difference between the non-energy harvesting MAC protocols and energy harvesting ones. Although more energy harvesting MAC protocols have been studied in the research, the studies are still limited to a few well-known MAC mechanisms. In contrast to the presented work in [19], the open issues and future research directions to improve the energy harvesting MAC protocols are highlighted in [20]. None of these related works have considered the actual energy consumption in the existing wireless communication technologies and the available amount of energy provided by each energy harvesting technique.

The authors in [21] first examined the amount of energy produced by different energy harvesting techniques and their characteristics. Then, they highlighted the requirements of the energy harvesting IoT systems. The presented classification in this work was accomplished by reviewing the principal characteristics of the energy harvesting MAC protocols the type of data transfer start point (sender, receiver, or sink initiated). The selected MAC protocols mainly operate based on the Carrier Sensing Multiple Access (CSMA) method, where the nodes listen to the shared medium before starting the transmission, and polling approach, which benefits from a poll frame to avoid collision frames in the transmissions. Similar to the aforementioned survey papers, the advantages and disadvantages of each selected protocol are explained. Also, their performance in terms of different KPIs is highlighted. In contrast to the two aforementioned survey papers, in [21], the authors studied both ambient and non-ambient energy harvesting MAC protocols. Moreover, in those papers, approaches such as hybrid access and cross-layer, which have recently attracted more attention due to their ability to optimize network performance, were not considered.

One of the latest classifications of energy harvesting MAC protocols is proposed in [22], where in addition to sender/receiver-initiated divisions are used by the aforementioned survey papers, approaches such as scheduled-based access protocols are included in this classification. Then, some energy harvesting MAC protocols’ functionality is explained based on the MAC layer requirements in IoT systems. Although this work includes a much more comprehensive number of energy harvesting MAC mechanisms than the previous surveys, the authors do not provide any information regarding the harvesters’ available energy, which is required to optimize the MAC layer operations.

Alongside the energy harvesting MAC protocol categorizations, some studies focus on a specific group or features of the IoT systems MAC protocols and then explain the available energy harvesting MAC protocol within the selected group. For instance [23,24], emphasized the importance of the Wake-Up Radio (WUR) concept in reducing the energy consumption of the IoT systems and studied the available WUR MAC protocols. Since the focus of these papers is WUR, no information is provided about the issues that arise with enabling energy harvesting techniques at MAC protocols. Another example that is presented in [25] classifies the MAC protocols in IoT systems based on a specific feature of these protocols and analyzes the MAC protocols operations regarding the selected feature. In this work, the authors consider wake-up/idle scheduling duration. Then, they explain the functionality of the most well-known energy harvesting MAC protocols within this classification, along with battery characteristics and conditions in IoT systems. Although this work provides a deep understanding of the MAC functionality that operates based on the wake-up/idle scheduling approach, there are other existing energy harvesting MAC protocols that cannot be explained through this classification. The authors in [25] accomplished a deep analysis of the estimation of the remaining energy in the batteries of wireless communication devices by presenting energy models of these batteries. However, the amount of available energy provided by different energy harvesters and actual energy consumption models based on various existing wireless communication technologies was not considered.

To the best of our knowledge, in the available state-of-the-art, the importance of the required amount of energy for the communication unit and the compatibility of the selected energy harvesting MAC protocols with the potential wireless communication technologies are not investigated. Also, the existing literature lacks a complete and sound classification covering all the most relevant existing energy harvesting MAC protocols in the literature, where not only the conventional approaches are studied, but also recent novel approaches are considered. Thus, in this survey paper, we fill this gap in the literature by investigating the mechanisms of the most relevant energy harvesting MAC protocols along with their benefits and drawbacks. Specifically, we first present a categorization for energy-aware MAC protocols based on the adopted channel access method. We also provide the actual amount of energy consumption in existing wireless communication technologies by providing a unified MAC-based approach. Finally, we study the available ambient and non-ambient energy harvesters in detail and investigate their compatibility with the existing wireless communication technologies.

## 3. Categorization of Energy-Aware MAC Protocols for IoT Systems

**Figure 3:**
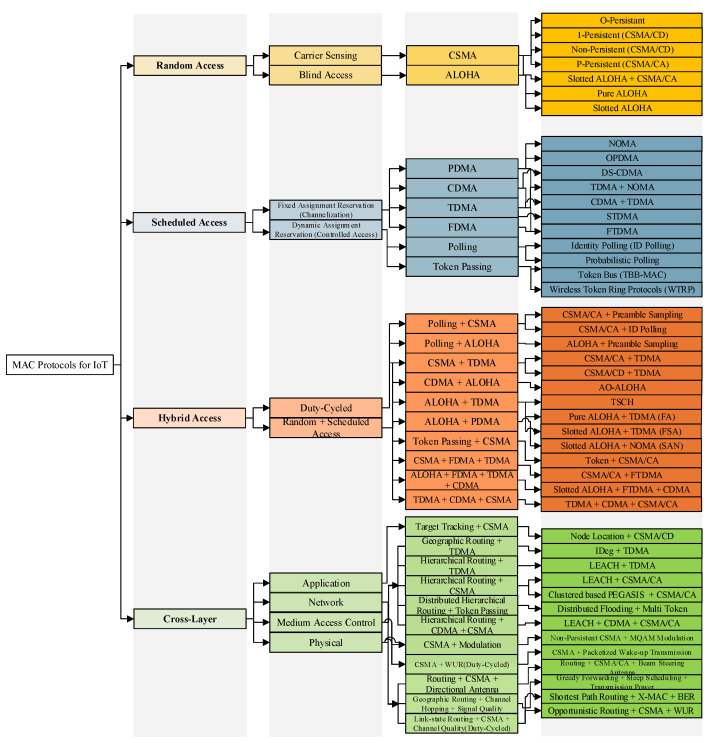
IoT systems Medium Access Control categorization.

According to the IoT protocol stack, the MAC layer is a sub-layer of the data link layer and is responsible for scheduling transmission or possible re-transmissions over the shared medium. In wireless communication technologies of IoT systems, MAC layer operations that provide adaptive channel access may face different problems such as collisions and idle listening (mostly in random access), or synchronization and unused idle slot (in scheduled access), which consume a significant portion of available energy of the IoT systems. Besides requiring different energy levels for MAC layer performance, wireless communication technologies are kept powered by conventional batteries. However due to the technological limitations of batteries, they may not perform for a long duration. Although the lifespan of the batteries can be increased by deploying energy harvesting technologies, due to the erratic and unpredictable nature of the provided energy, conventional MAC layer mechanisms are not compatible with these technologies. Thus they need modifications to integrate with energy harvesting techniques [21]. Since this paper focuses on the energy harvesting MAC protocols for IoT systems, this section describes how each MAC operates, together with the limitations of existing MAC protocols. These limitations can be understood with the help of a comprehensive categorization of energy-aware MAC protocols based on the performance and features of the protocols. Later in the document, Section 6 details how energy harvesting technologies are integrated within each energy-aware MAC protocol. This section provides an energy-aware MAC protocols categorization (cf. Figure 3).

### 3.1. Random Access

In this category, there is no coordinator to schedule the transmissions, and each node independently starts the transmission at any time. This category is divided into two subcategories: carrier sensing, blind access. Readers interested in further details of the random access category described in this subsection are referred to [26,27,28].

#### 3.1.1. Carrier Sensing

In the carrier sensing procedure, each node can sense the carrier signal of the other nodes of the network and decide whether to start the transmission or wait.

Carrier Sensing Multiple Access (CSMA): This sensing procedure is performed based on 1-Persistent and Non-Persistent, which are used in CSMA with Collision Detection (CSMA/CD) systems, or P-Persistent mechanism, which is used in CSMA with Collision Avoidance (CSMA/CA). In O-Persistent a supervisor updates the order of transmission for each node based on the ongoing transmissions.

#### 3.1.2. Blind Access

Since in this subcategory, the transmission procedure starts without sensing the shared medium, the probability of collision, and consequently, the energy consumption of the transmission procedure is higher than in the CSMA method.

ALOHA: This method is divided into Pure ALOHA and Slotted ALOHA mechanisms. Due to the slot definition in the Slotted ALOHA structure, the frame transmissions in the Slotted ALOHA mechanism are more energy-efficient from the end node perspective than the Pure ALOHA.

#### 3.1.3. Combination of Carrier Sensing and Blind Access

CSMA + ALOHA: This mechanism adds the collision avoidance feature of CSMA/CA to reduce the probability of frame collisions (Slotted ALOHA + CSMA/CA) and consequently reduce the wasted energy of re-transmitted frames.

### 3.2. Scheduled Access

Frame transmission in the scheduled access category occurs in a an organized manner, where all the nodes start transmissions at predefined slots or are controlled by a coordinator. Readers interested in further details of the scheduled access category described in this subsection are referred to [26,29].

#### 3.2.1. Fixed Assignment Reservation (Channelization)

In this subcategory, the shared medium is divided into a fixed amount of channel resources (slots of time/frequency/power/spread spectrum). Each node is only allowed to use the slots allocated to it and therefore does not contend to access the shared medium.

1. Code Division Multiple Access (CDMA): Before initiating the transmission, different codes are assigned to the nodes to encode their data.The most widely used mechanism of this method is Direct-Sequence CDMA (DS-CDMA), which reduces the total energy consumption of the network by using wide band signals with randomness that have lower interference compared to narrow band signals.
2. Time Division Multiple Access (TDMA): This method divides time into several periods, which itself is divided into a certain number of time slots. An advanced mechanism based on the TDMA method is Spatial Time Division Multiple Access (STDMA) that can reduce the energy consumption of the nodes by re-assigning time slots based on geographical locations/space, where the number of the unused slots is reduced by deactivating a certain number of slots which can save energy.
3. Frequency Division Multiple Access (FDMA): This method divides the medium into different frequencies, which are then assigned separately to each node. Since FDMA mechanisms such as Orthogonal FDMA (OFDMA) are non-energy efficient, it is out of scope in our current evaluation.
4. Power Division Multiple Access (PDMA): To share the power of the channel between nodes and avoid collisions, PDMA allocates specific transmission power to each node. The Non-Orthogonal Multiple Access (NOMA) intends to simplify the power division procedure while satisfying the QoS requirement of the transmission, co-channel interference cancellation, fairness improvement, and simultaneous successful frame reception [30]. The Orthogonal Power Division Multiple Access (OPDMA) [31] reduces the required energy for defining and assigning power slots to each node, and consequently, it decreases the network energy consumption.

#### 3.2.2. Combination of Channelization Methods

This subcategory combines the features of different channelization methods to improve energy efficiency, which leads to a more efficient resource allocation, transmission coordination, and more flexibility in terms of the traffic type and network size.

1. TDMA + PDMA: In this mechanism, nodes are divided into groups, and then a time slot is assigned to each group of nodes. Thus, the nodes within each group can start the transmission simultaneously [32].
2. TDMA + CDMA: In this method, time slots are assigned to different nodes which communicate through CDMA.With this combination, fewer time slots are required to have a successful frame transmission, which improves the performance of the network in terms of energy efficiency, flexibility, and scalability [33].
3. TDMA + FDMA: This mechanism, also called Frequency Time Division Multiple Access (FTDMA), in which, before initiating/allowing transmission, each node is assigned a specific time slot and appropriate frequency by the coordinator [34]. Thus, FTDMA reduces the number of the frame re-transmission which in return decreases the consumed energy of the network.

#### 3.2.3. Dynamic Assignment Reservation (Controlled Access)

In the dynamic assignment subcategory, coordination is achieved by a control message (poll, token), and only one node can start the transmission at a particular time.

1. Token passing: This method is divided into two mechanisms, Token Bus [35], where the token frame is passed in a probabilistic manner among a group of nodes enclosed in an area, and Wireless Token Ring Protocol (WTRP) [36], in which the nodes create a ring that token frame can pass through it only in one direction.
2. Polling: This method is divided into two mechanisms, Identity Polling (ID Polling) [37] and Probabilistic Polling [38]. In the first mechanism, a specific ID is assigned to each node. If the polling packet contains their ID, they start the transmission. If not, they have to wait for their turn. In the second mechanism, the polling packet contains the contention probability, which the coordinator assigns and allows each node to start the transmission according to a probability.

### 3.3. Hybrid Access

This category combines the benefits of the random (i.e., distributed nature, full channel utilization) and the scheduled access (i.e., contention-free for long frames) categories while diminishing their drawbacks. This category is divided into two subcategories: combination of random access and scheduled access, and duty-cycled.

#### 3.3.1. Combination of Random and Scheduled Access

A coordinator node schedules the timing for starting a random access-based data frame transmission. Thus, this method adapts to the network traffic conditions swiftly, optimizes the channel access method, and subsequently reduces the energy consumption of the network. Moreover, the mechanisms in the hybrid access category can guarantee the QoS, delay, and frame collision rate reduction. However, this strength in hybrid access may increase the level of MAC mechanisms complexity.

1. CDMA + ALOHA: The orthogonality feature of CDMA Slotted ALOHA [39] mechanism, makes the simultaneous transmissions possible with more efficient use of network resources and prevents degradation in the network’s performance.
2. ALOHA + TDMA: This combination is divided into three mechanisms, which are known as Frame ALOHA (FA), Frame Slotted ALOHA (FSA) [40], and Time Slotted Channel Hopping (TSCH) [41]. In these mechanisms, thanks to the contention-free feature of the TDMA, the number of frame re-transmission is reduced, and thus, the available energy of each node is conserved.
3. CSMA + TDMA: This combination includes CSMA/CA + TDMA [42,43,44], and CSMA/CD + TDMA [45] mechanisms. In these mechanisms, the reservation slot nature of TDMA provides a contention-free transmission for the mechanisms based on the random access category to avoid collision problems.
4. ALOHA + NOMA: One such mechanisms is the Slotted ALOHA-NOMA (SAN) [46] mechanism, which adds the MU feature of the NOMA to the slotted ALOHA mechanism to enhance the performance in terms of energy efficiency, low complexity, ease of implementation, and improved scalability.
5. Polling + CSMA: One of the mechanisms of this method is ID Polling + CSMA/CA [47], in which the coordinator defines the ID Polling period for each node by sending a specific polling frame to each node and then, each node only wakes up if it has a frame to send and transmits the frame based on the CSMA/CA mechanism.
6. Token passing + CSMA: A mechanism based on this method is known as Token + CSMA/CA [48,49], which is introduced as a multi-token-based approach with a random sleep scheduling structure.In this mechanism, several coordinators are defined to manage the transmissions based on their token frames.
7. CSMA + FDMA + TDMA: In CSMA/CA + FTDMA [50], the contention-free feature of both TDMA and FDMA is combined to conserve the energy, and the RTS/CTS handshaking method of CSMA/CA to reduce the hidden terminal issue.
8. ALOHA + FDMA + TDMA + CDMA: An example of this method is the Slotted ALOHA + FTDMA + CDMA [51], in which a coordinator node broadcasts time, frequency, and code slots to the nodes of the network. Then, each node randomly chooses a set of slots and is only allowed to start the frame transmission at these reserved slots. This pre-assignment of FTDMA and CDMA reduces collision probability and thus results in some energy saving at the node.
9. TDMA + CDMA + CSMA: In TDMA + CDMA + CSMA/CA [52] mechanism, the nodes are located inside the cluster (inter-cluster) and transmit short frames. In this mechanism, transmission power and times are controlled for each node; thus, the IoT systems’ energy efficiency is improved.

#### 3.3.2. Duty-Cycled

This subcategory is one of the main techniques to conserve transmission energy in IoT systems by adjusting the active and sleep duration of each node.

1. CSMA + Polling: This mechanism’s main target is to recognize the receiver, reduce the idle listening duration, and consequently reduce the total energy consumption of the network. This goal is achieved by applying a preamble sampling or preamble strobing approach, where each node transmits a low power preamble frame to announce the access point that it intends to start the transmission [25].
2. ALOHA + Polling: In this mechanism, the sender starts the transmission only a short duration before the receiver’s wake-up time based on the ALOHA method. Thus, the long preambles are reduced to shorter ones, which helps reduce the energy consumption of the network.
3. CSMA + TDMA: In this method, the data transmission procedure is started precisely after the receiver wakes up, making the listening duration shorter than previous approaches. Thus less energy is consumed during the listening period [25].

### 3.4. Cross-Layer

Since the network peripherals could be better managed by understanding the dynamics of each IoT protocol stack’s layer, in this category, two or three layers of the IoT protocol stack interact with each other simultaneously to optimize the performance of the network, especially in terms of energy consumption.

#### 3.4.1. Interaction between Application and MAC Layers

In this subcategory, the MAC layer interacts and exchanges information with the application layer to improve its mechanism. For instance, the application layer information, such as the QoS requirements or the application sensitivity to the network performance, can optimize the transmission scheduling process at the MAC layer.

Target tracking + CSMA: In a monitoring area, predicting the next location of a mobile node is known as target tracking, which is mostly done by its neighboring nodes. In this subcategory, the total energy consumption of the network is decreased by increasing the accuracy of the position estimation of the mobile node while sending some of its neighbors into sleep mode [53]. An example of this category is Node Location + CSMA/CD [54] mechanism, which intends to balance the trade-off between the QoS improvement and the network energy consumption.

#### 3.4.2. Interaction between Routing and MAC Layer

Since routing algorithms consume a considerable amount of energy, the interaction of an energy-efficient routing protocol with the MAC layer can improve network efficiency.

1. Geographic routing + TDMA: In this method, the geographic routing protocol generates the routing table based on the locations of the nodes. In IDeg-Routing + TDMA [55] mechanism, time slot assignments and routing tree generation occur simultaneously. Then based on the number of the routes to the same destination node, they are divided into single path or multipath scenarios. In multipath scenarios, the collision-free nature of the TDMA method and selecting the route with the highest amount of residual energy reduces the network energy consumption.
2. Hierarchical routing + TDMA: To reduce the energy consumption of the network and expand its lifetime, the Low Energy Adaptive Clustering Hierarchy (LEACH) routing approach is adopted in LEACH + TDMA [56] method, in which the network is divided into several clusters. Each cluster head is selected randomly and based on the remaining energy of the node and its distance to the base station.
3. Hierarchical routing + CSMA: This method includes two mechanisms, LEACH + CSMA/CA [57] and Power Efficient Gathering in Sensor Information Systems (PEGASIS) + CSMA/CA [58]. In the first mechanism, due to the sleep duration added to the CSMA/CA, the energy consumption of the nodes in LEACH + CSMA/CA is lower than LEACH + TDMA. In the PEGASIS mechanism, to reduce the energy consumption of the network, all the nodes go to the sleep state unless they have a frame to send or if they are going to receive a frame.
4. Distributed hierarchical routing + Token passing: One example of this method is an energy-efficient cluster-based routing protocol that interacts with a token passing method. The target of Distributed Flooding + Multi Token [59] mechanism is that the network continues to operate even if some nodes are disconnected from the network and conserve the network energy consumption.
5. Hierarchical routing + CDMA + CSMA: An example of this method is LEACH + CDMA + CSMA/CA [60,61], in which the inter-cluster nodes are assigned to a certain number of time slots based on their available energy levels, which can schedule the sleep state duration of each node. For these reasons, some part of the energy budget of the network is saved, and the network lifetime is expanded.

#### 3.4.3. Interaction between MAC and Physical Layers

In this method, several parameters of the physical layer, such as power and sub-carrier allocation strategies, antenna cooperation, and beam-forming techniques, are used to enable the energy-efficient scheduling transmission in the MAC layer.

1. CSMA + Modulation: An example of this method is an interaction of an adaptive modulation with a multi-channel CSMA, known as CSMA + M-Ary Quadrature Amplitude Modulation (MQAM) [62]. In this mechanism, the modulation scheme information, along with an adaptive back-off probability, can reduce the delays due to the re-transmissions and thus save the energy budget of the network.
2. CSMA + WUR: This method proposes a CSMA-based mechanism with two different approaches for ultra-low power networks. The first one reduces the energy consumption due to the overhearing issue by continuously packetizing the wake-up transmissions. The second one operates based on the energy existence on the channel and reduces the size of the receive check [63].

#### 3.4.4. Interaction between Routing, MAC and Physical Layers

In this method, the routing and physical layer innovations can assist the MAC layer to operate in an energy-efficient manner [64].

1. Routing + CSMA + Directional antenna: An example of this method is Routing + CSMA/CA + Beam Steering Antenna [65], in which to conserve the energy budget of the network, the CSMA method adjusts the wake-up/sleep scheduling duration of the radio transceiver to the traffic load adaptively and makes the transmissions more energy-efficient. This mechanism benefits from the advantages of directional antennas (where the antenna only radiates in a narrower geographical area), making simultaneous transmissions possible.
2. Geographic routing + Channel hopping + Signal quality: A mechanism that belongs to this method is Greedy Forwarding + Sleep Scheduling + Power Transmission [66,67], in which according to the information that is provided by the routing protocol and the amount of transmission power, the MAC layer decides the sleep duration of each node; thus, it can reduce the network energy consumption.
3. Link-State routing + CSMA + Channel quality: The (Shortest Path Routing + CSMA + BER) [68] is an example of this method, which reduces the energy consumption of the network through an estimation of the Bit Error Rate (BER) and updating the information from the network layer (distance table) along with the short adaptive duration of sleep mode.
  The last mechanism refers to the interaction of a duty-cycled-based method and an opportunistic routing protocol. This mechanism applies different estimation techniques such as the Expected Duty Cycled Wakeups (EDC) and Energy-Centric Data Collection with Anycast in Duty-Cycled (EDAD). It uses the information provided from opportunistic routing protocols to approximate the number of required wake-ups for transmitting a data frame. For this reason, this mechanism schedules the idle listening and wake-ups more precisely and thus reduces the energy consumption of the network [69,70,71].

## 4. IoT Technologies and Energy Models

In recent years, IoT communication technologies connect new approaches and concepts to meet energy efficiency requirements. In order to define the IoT requirements in terms of energy efficiency, available tools like energy models or empirical energy measurements have been used in the literature.

This section first summarizes each IoT communication technology regarding its available MAC layers and energy consumption characteristics. The available energy models in the literature are then presented and categorized based on their wireless communication technologies. In the end, this section is summarized by providing a comparison of wireless communication technologies and reviewing their applicability for IoT systems in terms of energy consumption. For this reason, the total power consumption of each technology is modeled based on the different states of its MAC layer. The parameters from the equations used in the energy consumption analyses for IoT technologies are explained in Table 1.

### 4.1. Wireless Local Area Network (WLAN)

The Institute of Electrical and Electronics Engineers (IEEE) 802.11 technology was designed based on a random access mechanism (CSMA/CA), which is an energy-consuming protocol [72]. The reason for that is the collision avoidance functionality of this protocol, which keeps stations awake (in active mode) to listen to the channel for a certain duration before attempting to transmit [73]. To cater to this drawback, a power management mode was introduced to IEEE 802.11 standard [74]. In this technology, the total energy consumption is obtained through the consumed energy within the different states of the communication, where the consumed energy of each state is the multiplication of the power consumption of that state to its corresponding duration. The energy consumption model for IEEE 802.11 technology is obtained through Equation (1), defined in [75].

(1) $$E_{\mathrm{Total}}=T_{\mathrm{Rx}}.P_{\mathrm{Rx}}+T_{\mathrm{Tx}}.P_{\mathrm{Tx}}+T_{\mathrm{Id}}.P_{\mathrm{Id}}+T_{\mathrm{Sl}}.P_{\mathrm{Sl}}$$

The IEEE 802.11 standard group has introduced in recent years different amendments that aim to satisfy the IoT systems requirements. Within these amendments, the original channel access method has been changing through the technical definition of each amendment, looking for better performance in IoT systems. Below, the most relevant energy models for the IoT-compatible IEEE 802.11 amendments are described.

#### 4.1.1. IEEE 802.11ah

Similar to the legacy IEEE 802.11, the channel access method of IEEE 802.11ah is based on CSMA/CA. However, additional features to the MAC layer such as hierarchical Association IDentifiers (AID), group sectorization, Restricted Access Window (RAW), Relay Access Point (Relay AP), bi-directional Transmission Opportunity (TXOP), and Target Wake Time (TWT) [76], make IEEE 802.11ah acceptable for IoT systems with a large number of devices deployment (by reducing the contention ), and low power communications. Due to the equality of the channel access method of this amendment and IEEE 802.11 standard, the total energy consumption of this technology is also obtained through Equation (1). The IEEE 802.11ah energy model was first introduced by Raeesi et al. [77], where the sleep duration is extended, and power consumption of transmission state is reduced by utilizing short beacon frames, short MAC header, etc. Thus, the total energy consumption is reduced. This energy model was later reformulated by Bel et al. [78] to consider TIM and page segmentation scheme.

Along with the analytical models, empirical power consumption analyses have been performed based on real hardware measurements included in the Orinoco Wireless Fidelity (WiFi) card data sheet [79] and smart grid IEEE 802.11ah chip designed in [80]. The analytical models do not consider the energy consumption of hardware components such as the microcontroller, whereas energy models based on real measurements do so.

#### 4.1.2. IEEE 802.11ax

The channel access method of this technology adds OFDMA on top of CSMA/CA.A MAC feature that makes IEEE 802.11ax a suitable technology for dense environments is the Basic Service Set (BSS) coloring [81]. Moreover, in this technology, energy efficiency can also be achieved through approaches such as microsleep, TWT, and Opportunistic Power Save (OPS) [82].

According to the channel access mechanism of IEEE 802.11ax technology, the energy model of this technology includes the basic four states of Equation (1). An OFDMA-based Hybrid Channel Access (OHCA) for uplink MU transmissions is introduced in [83], and an energy model based on the MU power-saving mode is proposed in [84]. Through this model, the authors showed that, by defining a certain sleep duration for the uplink flow, it is possible to save a significant amount of power. The total energy consumption for the power saving mode is obtained through Equation (2).

(2) $$E_{\mathrm{Total}}=T_{\mathrm{Rx}}.P_{\mathrm{Rx}}+T_{\mathrm{Tx}}.P_{\mathrm{Tx}}+T_{\mathrm{Sl}}.P_{\mathrm{Sl}}$$

According to Equation (2), for uplink transmissions, the station only wakes up from deep sleep mode when it wants to receive or transmit frames. Thus, in power saving mode, the idle mode is removed from the total energy consumption Equation (1).

#### 4.1.3. IEEE 802.11ba

This amendment aims to balance the trade-off between low latency and low power states (1mW) in devices [85,86,87] while being backward compatible [88]. This amendment works on WUR, whose implementation is based on a Wake-up Transmitter (WuTx) and Wake-up Receiver (WuRx). Since the WuRx is a very low power consumption radio and the primary radio wakes up on-demand, the power consumption during idle mode decreases significantly [88].

The channel access method IEEE 802.11ba is the Enhanced Distributed Channel Access (EDCA) based on CSMA/CA, and power-saving mode is fulfilled through the WUR. The total energy consumption of IEEE 802.11ba is modeled in [89], where a dynamic hybrid WLAN communication model for IoT devices is considered. In this model, MU operation (IEEE 802.11ax) and WUR (IEEE 802.11ba) are taken into account. According to the proposed model presented in [89], the total energy consumption in each station is achieved through Equation (3).

(3) $$E_{\mathrm{Total}}=T_{\mathrm{Rx}}.P_{\mathrm{Rx}}+T_{\mathrm{Tx}}.P_{\mathrm{Tx}}+T_{\mathrm{Id}}.P_{\mathrm{Id}}+T_{\mathrm{Wu}}.P_{\mathrm{Wu}}+2T_{\mathrm{Sw}}.P_{\mathrm{Sw}}$$

Presenting an efficient scheme for the wake-up receiver, while considering the coexistence with IEEE 802.11 legacy, is becoming an attractive topic [88,90,91,92,93], where some of these energy models are proposed based on the AS3933 chip by Austria Microsystems.

### 4.2. Low-Power Wide Area Network (LPWAN)

Unlike the above IEEE 802.11 amendments, LPWAN technologies support longer communications with extremely low bandwidth, low power consumption, and constrained duty-cycles (how frequently an end-device transmits data) [94]. These features make LPWAN technologies more attractive for IoT than other long-range communication technologies. Long-Range Wide Area Networks (LoRaWAN), Sigfox, and Narrow-Band IoT (NB-IoT) are described next as LPWAN examples.

#### 4.2.1. LoRa

The basic channel access method is a simple random channel access mechanism (Pure ALOHA/Slotted ALOHA), which simplifies this technology at the protocol level [95]. However, to reduce the power consumption in long-range communication, the MAC layer of this technology has been modified by adding sleep mode (CSMA feature) at its protocol level [96,97].

To the best of our knowledge, the basic energy models for LoRa technology are designed based on the real Class A device characteristics to the date of this paper. An energy consumption analysis and its model design for the most common LoRa transceiver chip, SX1272, are presented in [98,99]. The accuracy of the proposed energy model, which mimics the energy consumption of the LoRaWAN Class A device, is evaluated through simulation in Network Simulator-3 (NS-3) [99]. Thus the total energy consumption of a simple data transmission procedure can be derived from Equation (4).

(4) $$E_{\mathrm{Total}}=T_{\mathrm{Rx}}.P_{\mathrm{Rx}}+T_{\mathrm{Tx}}.P_{\mathrm{Tx}}+T_{\mathrm{Sl}}.P_{\mathrm{Sl}}$$

An energy consumption estimation based on the different states of the LoRaWAN hardware is proposed in [100] through Equation (5). Since this model is formulated based on a real sensor node, some parameters such as a wake-up transceiver, microcontroller, and data measurement are added to Equation (4). According to the authors, this model is applicable for Class A end devices.

(5) $$\begin{array}{c} E_{\mathrm{Total}}=E_{\mathrm{Rx}}+E_{\mathrm{Tx}}+E_{\mathrm{Sl}}+E_{\mathrm{Wu}}+E_{m}+E_{\mathrm{Mcu}}+E_{\mathrm{Wut}} \end{array}$$

#### 4.2.2. Sigfox

The channel access method in Sigfox is known as Random Frequency and Time Division Multiple Access (RFTDMA). To provide a simple and low power consumption at the protocol level, this technology limits the number of data transmissions [101] and does not use packet synchronization and beacon frames [72].

According to the authors in [102], the total energy consumption of a bidirectional Sigfox-based communication for the MKRFOX1200 device can be modeled based on the different states of the transaction, which includes wake-up, transmission, listening, reception, cool down, and sleep states. According to this model, the total energy consumption of a MKRFOX1200 device is obtained through Equation (6).

(6) $$E_{\mathrm{Total}}=T_{\mathrm{Wu}}.P_{\mathrm{Wu}}+T_{\mathrm{Rx}}.P_{\mathrm{Rx}}+3.T_{\mathrm{Tx}}.P_{\mathrm{Tx}}+2.T_{\mathrm{Id}}.P_{\mathrm{Id}}+T_{\mathrm{Cd}}.P_{\mathrm{Cd}}+T_{\mathrm{Sl}}.P_{\mathrm{Sl}}$$

Moreover, in [102] an analytical model for energy consumption of data delivery and battery lifetime of the MKRFOX1200 Sigfox device is proposed. The novelty of this work is the impact consideration of the frame losses.

#### 4.2.3. NB-IoT

This technology reduces the power consumption by using different power saving mechanisms such as Handling Re-transmission (HARQ), Extended Discontinuous Reception (eDRx), and Wake-up (Wu) signal. The eDRx reduces the power consumption by keeping the device in an inactive mode for a long duration (deep sleep mode), whereas Wu signal is sent during the idle mode to wake-up the main receiver [103].

Although in NB-IoT technology, different channel access methods are deployed for uplink and downlink communications (SC-FDMA, OFDMA), a single energy model is valid for both uplink and downlink communications, where the energy consumption of transmission mode can be defined as an uplink or downlink flow. This model is defined in [104], where four different communication modes (wake-up and connect, transmission, disconnect and sleep/idle) are considered. Equation (7) expresses the total energy consumption of this technology.

(7) $$E_{\mathrm{Total}}=E_{\mathrm{Wu}}+E_{\mathrm{Tx}}+E_{\mathrm{Disconn}}+E_{\mathrm{Id}}$$

In the work presented by [105], two different methods are defined for each communication flow. In this model, the authors utilized power-saving mode for uplink and eDRX mechanism for downlink communications (sleep/idle mode extension) to reduce the total energy consumption. The authors showed that their proposed energy model is able to prolong the battery life over 12 years.

### 4.3. Radio Frequency Identification (RFID)

Compared to the LPWAN technologies, which provide long-range communication, RFID and Near-Field Communication (NFC) are suitable for shorter-range applications.

The high-level classification of RFID technology divides it into passive and active.

Passive technology tags are kept powered through a passive technique such as energy harvesting methods or backscattering. One of these technologies is the Electronic Product Code Class 1 Generation 2 (EPC Gen2) [106]. Passive tags are small, cheap, and ultra-low power consumption, these features making them suitable for massive deployment environments and hard to reach use cases. However, this technology suffers from a low communication range (10 m) [107].

Active technology refers to the devices that require power supply on tags such as a battery. This technology is not as popular as passive RFID. The active technology can transmit stronger signals and, in consequence, provide a more extended range of communication (100 m) [108]. However, compared to passive tags, they are large and expensive (due to their on-board batteries).

Due to the random nature of the channel access method in this technology (Slotted ALOHA or FSA), transmission and reception states are considered to define the energy model. The proposed energy model in [109] is based on an energy-aware ALOHA channel access, where the consumed energy per tag is obtained through Equation (8).

(8) $$\begin{array}{c} E_{\mathrm{Total}}/n=E_{\mathrm{Rx}}/n+E_{\mathrm{Tx}}/n \end{array}$$

### 4.4. Wireless Personal Area Network (WPAN)

In contrast to the above wireless technologies, the WPAN technologies are applicable only for personal network applicationswhere short-range and low power consumption communications are required. Among WPAN technologies, Bluetooth Low Energy (BLE-Bluetooth 4.0) and Zigbee (IEEE 802.15.4) are the most energy-efficient and cost-effective ones. Thus, they are the most adopted WPAN standards for IoT use cases [110].

#### 4.4.1. BLE

The BLE-based device is always in sleep mode and wakes up only for short periods based on its channel access method (TDMA). This wake-up period is called connection state [72]. Based on the proposed energy model in [111], during these periodic wake-up intervals, a short delay is defined as the Interframe space (Ifs). This delay is the time interval between transmission and reception in each connection. The total energy consumption of the slave connection is expressed through Equation (9).

(9) $$E_{\mathrm{Total}}=E_{\mathrm{Wu}}+E_{\mathrm{Rx}}+E_{\mathrm{Tx}}+E_{\mathrm{Ifs}}+E_{\mathrm{Mcu}}$$

The energy model presented in [111] is designed based on the BlueGiga BLE112 hardware module (based on TI’s CC2540 System-On-Chip). Since this model is based on real hardware, some parameters such as energy consumption in microcontroller and Ifs are also taken into account.

#### 4.4.2. Zigbee

Similar to the IEEE 802.11 legacy, the basic channel access method of this technology is based on the CSMA/CA mechanism [72]. Since it is a low data rate technology, devices usually stay in sleep mode and only wake up for short periods to send data, reducing power consumption. Compared to the IEEE 802.11ah/ax, Zigbee is a low complex and low power consumption (about four times less) technology, which prolongs the battery lifetime while it provides less coverage range [112].

The proposed energy model in [113] is designed based on the random nature of the channel access method, where due to the three aforementioned characteristics, the number of collisions and consequently re-transmissions are reduced. Since the channel access method in Zigbee and IEEE 802.11 standard is the same, the total energy consumption for this technology is also obtained based on Equation (1).

The aforementioned wireless communications are just a few technologies that can be considered the potential communication technologies for IoT systems. Other potential communication technologies can be listed as Z-Wave, Weightless SIG, Wireless Highway Addressable Remote Transducer (WirelessHART), THREAD, ANT+, Long Term Evolution for Machines (LTE-M), and Extended Coverage Global System for Mobile communication (EC-GSM). Readers interested in further details of different LPWAN technologies are referred to the papers [94,98].

### 4.5. Comparison of Communication Technologies and Their Suitability for IoT Regarding Energy Consumption

**Figure 4:**
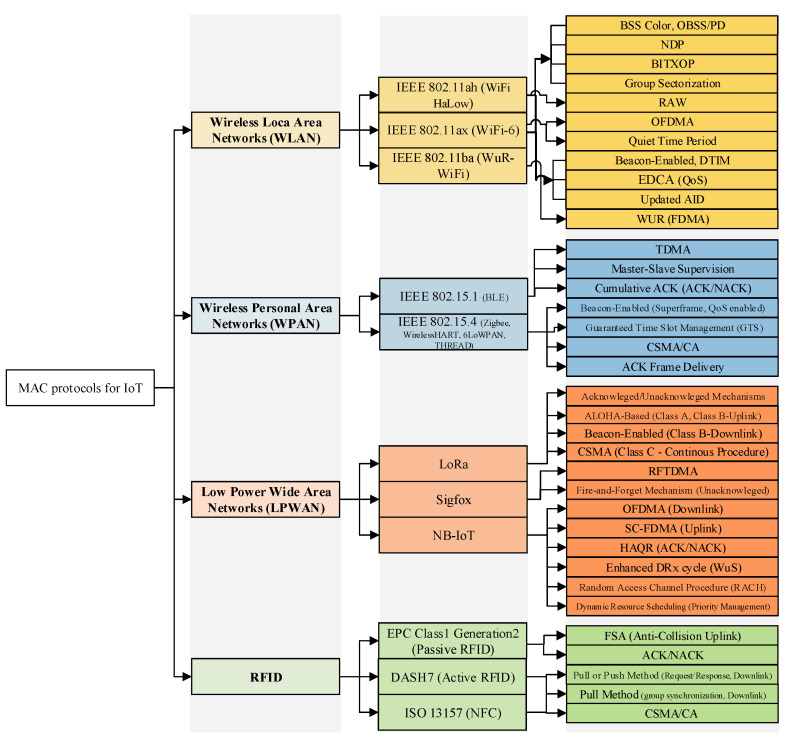
Overview of the MAC protocols of the current IoT technologies.

Table 2, which is built based on the references [72,76,88,114], provides a brief comparison of the technologies mentioned above in terms of the most relevant aspects regarding the IoT paradigm. Then, the IoT-related MAC features of each potential communication technology are listed in Figure 4.

For instance, in the LPWAN family, NB-IoT is a license-based standard, while LoRa and Sigfox work on the unlicensed Radio Frequency (RF) spectrum, and these technologies support communications with a long-range and low data rate. For this reason, they are mostly deployed in IoT applications such as smart cities and industrial automation. Other than LPWAN technologies, WLAN technologies benefit from the characteristics and features of the CSMA method to manage the collision frame during the transmission procedure and provide services such as QoS and QoE. For example, to support QoS, WLAN technologies support EDCA based on their MAC layer functionality, whereas this is not provided in LPWAN proprietary technologies (cf. Figure 4). In contrast to LPWAN and WLAN families, BLE technology which belongs to the WPAN family is the most widely deployed in IoT healthcare applications due to the collision-free characteristic of its MAC layer and its low power consumption communications. However, since the WPAN family is applicable for short-range transmissions, it may not be practical in IoT applications that require long-range such as smart transportation.

According to the power consumption values of the different communication states of IoT technologies (cf. Table 3), and the limitations of each wireless communication technology, some of these technologies may be more appropriate for IoT implementation.

In comparison with IEEE 802.11 legacy, which is a power-hungry technology regarding IoT, IEEE 802.11ah and IEEE802.11ba have been able to reduce energy consumption (about 7.5 times for IEEE 802.11ah and 5 times for IEEE 802.11ba less than legacy) by introducing specific modifications [76,87]. Although the aim of IEEE 802.11ax is dense deployment (not exclusively for IoT), it can reduce the energy consumption of the network devices up to 1.5 times less than legacy by extending the duration of sleep mode [82]. However, since IEEE 802.11ba is still under development and IEEE802.11ax has not reached sufficient technical maturity yet, these technologies require more time to be well adapted for IoT applications. Since LPWAN technologies provide low energy consumption (about 7 times less than WLAN technologies) and very long coverage range (up to 100 km in case of NB-IoT) [122] communications compared to the above wireless technologies, these technologies include a wide range of IoT applications from smart buildings to industrial automation. However, the main drawback of LPWAN technologies is their low data rate. Although energy consumption by WPAN communications is lower than the aforementioned WLAN technologies (10 times less) [116,120], due to their frequency band (2.4 GHz), they suffer from interference, resulting in a loss of communication reliability. Thus, WPAN is only appropriate for smart home and wearable devices. Moreover, EPC Gen2 RFID [106] is one standard allowing passive communications, which is suitable for IoT applications in the supply chain system, with a limited communication range.

Based on the available state of the art on IoT wireless communication technologies, LPWAN technologies and IEEE 802.11ah are promising ones. Nevertheless, the limited battery lifetime of these technologies may represent an issue for specific IoT scenarios. For instance, those requiring high availability or low maintenance cannot afford frequent battery replacement. Hence, techniques to prolong battery lifetime, like energy harvesting, may improve different IoT applications.

## 5. Energy Harvesting Solutions for IoT Technologies

Energy harvesting systems are applied to the IoT paradigm to prolong the battery life time and make these systems more energy-efficient. The classification of energy harvesting mechanisms is based on their inherent characteristics such as, scalability, maintainability, ability to improve IoT devices life time, form factor, capacity, and sustainability. The energy harvesting mechanisms are fed by environmental and non-environmental energy sources. The former includes sun radiation, wind and water flows, geothermal, within others, whereas the latter refers to RF signals and mechanical forces.

In this section, first the most relevant IoT related features of energy harvesting systems are highlighted. Then, the structure and the functionality of those energy harvesting technologies are explained. In the end, an investigation of the compatibility of energy harvesters and the aforementioned wireless technologies is provided.

### 5.1. IoT Energy Source Characteristics

One of the main difficulties that IoT systems face is the limited sources of energy to keep devices powered, which is traditionally provided by batteries. Since IoT systems include thousands of devices, frequent replacement of their batteries or finding the failed ones, require time and human intervention, which increase the cost of maintenance. This issue becomes worse when the devices are located in hard-to-reach areas or mobile locations. The above battery shortcomings as energy source for IoT systems, bring energy harvesting technologies into consideration.

To continuously keep IoT systems powered, energy harvesting technologies harvest energy from the surrounding environment, which may provide longer lifetime and lower maintenance operations. Additionally, most of the energy sources in energy harvesting technologies can provide the required power for wireless communications, which makes these technologies scalable to various IoT applications and services. In contrast to battery disposal, which has negative effects to the environment [123], energy harvesting technologies can alleviate these harmful effects and move towards sustainable IoT systems. The above advantages of energy harvesting technologies over batteries can make IoT systems more feasible and cost-effective.

Table 4 highlights the characteristics of the main energy sources that feed harvesting systems. The main energy harvesting techniques for IoT systems are described next.

### 5.2. Suitable Energy Harvesting Technologies for IoT

**Figure 5:**
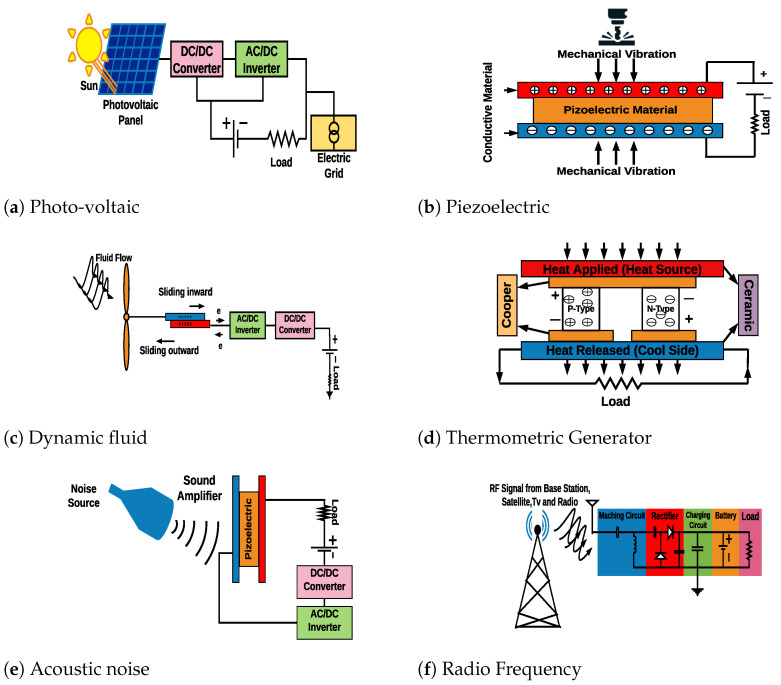
Existing energy harvesting mechanisms according to their source of energy.

In this section, we briefly highlight the suitable energy harvesting techniques that can be utilized in IoT devices. These methods can be fed by ambient or non-ambient sources. The illustration of each mechanism is shown in Figure 5.

#### 5.2.1. Solar-Based Energy Harvester

The high power density feature of the solar cell, makes it a suitable power unit technique for IoT applications such as smart agriculture and smart city. The solar or photo-voltaic cells absorb the energy from a natural or artificial source of light (sun or fluorescent light), and then convert it to electric current. The converted energy is conducted into two metals in the top and bottom of the cell, and is usually stored in a super-capacitor or a battery to keep IoT devices [124] powered. Based on the type of the source of light, solar cells can be indoor or outdoor, which vary in size. These cells can be as large as a solar panel (integrated cell) or a small thin-film (Dye-Sensitized). Depending on the amount of solar or fluorescent light radiation, the power density varies from 10 µW/${\mathrm{cm}}^{3}$ to 100 mW/${\mathrm{cm}}^{3}$ [125,126]. However, due to the size of the cell, stochastic characteristic of the energy source, and the amount of wasted energy (significant amount energy is turned into heat or is reflected by the surface of the cell), it is not a feasible method for some IoT applications such as wearable devices [127]. The simplified structure of a photo-voltaic module is shown in Figure 5a.

#### 5.2.2. Mechanical-Based Energy Harvester

In contrast to solar cells, mechanical harvesters are smaller in size and provide a high power density. For these reasons, they are widely used in IoT applications (from wearable devices to monitoring). The mechanical energy is divided into two groups, kinetic and potential, where the former is generated through motion, vibration, pressure and human activity, and the later one is generated based on the position of the energy source. The mechanical energy harvesters are designed based on three different methods, electromagnetic, electrostatic and piezoelectric. Within these methods, the piezoelectric energy harvesters are light-weight and cost-effective and provide high output voltage, energy density, and capacitance, which make them more suitable for IoT applications [128]. The piezoelectric energy harvesters operate based on the piezoelectric material where the crystal ionizes under a certain strain, and is able to convert kinetic energy to electrical energy. The piezoelectric modules are known as cantilever beam, circular diaphragm, cymbal, and stacked structure [128]. Based on the type of the piezoelectric material and the amount of energy source, these modules provide a wide range of power density from 0.021 µW/${\mathrm{mm}}^{3}$ to 2 W/${\mathrm{cm}}^{3}$ [10,129]. However, these materials are frangible, easy to break, and can be toxic [10]. Figure 5b illustrates the simple structure of the piezoelectric energy harvester.

#### 5.2.3. Dynamic Fluid-Based Energy Harvester

Compared to the solar cells, dynamic fluid energy harvesters have lower power density and have more limitations regarding the installation site. The dynamic fluid energy source is divided into two main types, wind, and water. The most common energy harvester in this category is the turbine. According to the general structure of the turbine, the blades are connected to a shaft that can spin the generator by its rotation. Microturbines (windmill [130] and wind-belt [131]) were designed to make the wind turbine suitable for IoT applications regarding their scale, however, the efficiency of these turbines is decreased by reducing the size of the blades [132]. Moreover, hyper-power turbines that are available in different scales are used for the flowing water energy source. Since this method is flexible in size, pollution-free, and has a continuous source of energy, it is feasible for IoT applications [125]. According to the scale of the harvesters and their installation site, they provide a power density from 1 mW/${\mathrm{cm}}^{2}$ to 41.2 mW/${\mathrm{cm}}^{2}$ [10,133,134]. Nevertheless, turbines have some limitations, such as feasibility only in open, windy, or near the sea areas. Figure 5c shows the basic structure of a dynamic fluid energy harvester.

#### 5.2.4. Thermal-Based Energy Harvester

Compared to the above energy harvesters, thermal energy harvester modules provide a range of power density between solar cells and turbines. Thermal energy harvesters include geothermal, waste heat from the industrial sector, solar heat, or even the human body [125]. The thermocouple or Thermometric Generator (TEG) is a widely known example of thermal energy harvester. It is made of two different metals or semiconductors, which generate a voltage, based on the temperature difference between their two junctions [129]. These harvesters have a long life and low maintenance, however, their low efficiency (5–11%) has prevented them from being widely used in IoT applications [114,125]. According to the type and the reflected heat of the TEG, its power density varies between 40 µW/${\mathrm{cm}}^{2}$ and 50 mW/${\mathrm{cm}}^{2}$ [10]. Figure 5d illustrates a typical model of a TEG made of semiconductors.

#### 5.2.5. Acoustic Noise-Based Energy Harvester

Compared to the above energy harvesters, the lowest power density is generated by the acoustic noise harvester. The energy source of acoustic noise is based on sound waves (longitudinal, transverse, bending, hydro-static, and shears waves) and vibration [125]. According to the functionality of the acoustic noise harvester, they are divided into three main types, Helmholtz resonator-based, quarter-wavelength resonator-based, and acoustic metamaterial based techniques [135]. To generate power from noise waves, first, the noise is directed into the barrier and vibrates in the resonator. Then the converters change the resonance into electricity, which can be stored in the super-capacitors or batteries [135]. Among the aforementioned energy harvesters, acoustic noise harvesters usually provide the lowest power density (up to 960 nW/${\mathrm{cm}}^{3}$), and there are scarce environments with the required level of acoustic noise. Hence, it is only a feasible method for powering up some IoT applications such as infrastructural monitoring [125]. Figure 5e illustrates the basic structure of an acoustic noise energy harvester.

#### 5.2.6. Radio Frequency-Based Energy Harvester

After acoustic noise harvester, Wireless Energy Harvesting (WEH) methods generate the lowest power density among the aforementioned techniques. RF signals are divided into two main groups, dedicated and radiated signals. The former group relies on RF transmitters included in the same IoT system, which usually have predictable features [136], whereas the latter group includes ambient RF signals that are radiated from other sources like TV, GSM, WiFi, microwave, ovens, or radar among others. The fundamental parts of an RF energy harvester are known as the receiving antenna, matching circuit, peak detector, and voltage elevator, which are shown in Figure 5f. The combination of the peak detector and voltage elevator is usually named rectifier and the RF energy harvester is named rectenna. In principle, RF signals are received by an antenna. Then in the matching circuit, the voltage is amplified by matching the antenna impedance to the rectifier circuit. Finally, the rectifier which is a part of Alternating Current/Direct Current (AC/DC) converter, captures the AC signal and converts it to a DC signal [126]. It is possible to store the energy by adding a capacitor (rechargeable battery) to the RF energy harvesting module or power-up, for instance, a passive RFID tag [114]. Due to the simplicity, availability, and easy to implement features of RF signals, WEH methods are a promising solution for IoT systems [137]. However, since the efficiency of RF energy harvesting systems depends on the amount of captured power and AC/DC conversion effectiveness, they are not practical in the rural areas [10]. Based on the physical characteristics and installation site of the WEH, the amount of generated power density by rectenna can vary from 0.1 µW/${\mathrm{cm}}^{2}$ to 300 µW/${\mathrm{cm}}^{2}$ [129,138].

### 5.3. Compatibility between Communication and Energy Harvesting Technologies

The available amount of energy that is harvested from ambient and non-ambient energy sources by each existing energy harvester is listed in Table 5. Generally speaking, among the aforementioned energy harvesters, solar cells and turbines can provide more power density, however, their large scale and availability are their main drawbacks, and make them mostly suitable for outdoor IoT applications [139]. Moreover, piezoelectric materials that are widely used as mechanical and acoustic noise harvesters suffer from brittleness. Since thermal energy source is independent of environmental conditions and uses a simple harvester system to scavenge the energy, it can be well adopted in different IoT applications such as healthcare (wearable devices) [140]. However, the main drawback of the thermal energy harvester is its low efficiency. Different IoT applications such as smart city and healthcare can benefit from RF harvesting systems, where the RF signals are converted to electricity to keep those devices powered. Nevertheless, compared to solar cells and turbines, RF harvesters provide lower power density, and RF signal strength depends on the distance between its harvester and the signal source.

The intersection of the available harvested energy from existing energy harvesting technologies (cf. Table 5), and the power consumption IoT technologies analysis from Section 4, defines suitable combinations of these technologies. Due to its low-power consumption, LPWAN technologies like LoRa can benefit from a wide range of energy harvesting technologies like solar panel or thermocouple, making it a promising IoT technology. Another long-range wireless communication technology like IEEE 802.11ah, although having higher power consumption, can benefit from more powerful energy harvesting technologies like solar panel or wind force for outdoor use cases.

Different examples in the literature show that it is possible to provide a certain percentage of the required power for the IoT systems by means of energy harvesting technologies. This amount of harvested energy adds to the battery and prolongs its operational lifetime. For instance, the waste heat from a central heating installation, can extend the operational lifetime of the batteries in a monitoring system powered based on a WiFi communication technology [141]. Based on the size of the solar cells and the amount of illumination of the sun, solar energy harvester modules can keep devices powered by extending the battery’s lifetime, which operate under LPWAN technologies [139]. Due to the low power consumption of the WPAN technologies, they can benefit from ambient RF [142] or thermal energy harvesting from the human body [143]. Moreover, a dedicated RF source can keep low-power RFID applications powered [144]. Experimental results combining the existing literature, and the available amount of energy that can be provided by different energy harvesters, are summarized in Table 6. Thus, according to the available power density of these energy harvesting systems (cf. Table 5), they can prolong the battery’s lifetime in IoT communication technologies (cf. Table 3), as detailed in Table 6. For instance, a TEG harvester can add about 10% of the IEEE 802.11ah WiFi required power to the batteries of the system, and thus, prolong their lifetime.

Besides the limited harvested power and the hardware related limitations of the energy harvesters, there is a need to improve the IoT communication technologies in terms of energy consumption. In the design procedure of potential wireless communications for IoT, the requirements of energy harvesting systems and their constraints are not well taken into account. Thus, there is a need to change the legacy protocols to accommodate the unpredictable behavior of energy harvesting sources. This requires a comprehensive review of relevant existing energy harvesting MAC protocols in the literature.

## 6. Energy Harvesting MAC Protocols

In Section 3, we provided a comprehensive classification of the recent existing energy-aware MAC mechanisms in the literature for IoT systems. However, since these mechanisms are not designed based on the intermittent nature of the energy harvesting energy sources, they may not provide sufficient energy for these techniques. To fill this gap and enable the integration of the energy harvesting techniques with existing communication technologies, various energy harvesting MAC protocols are proposed in the literature. However, since these protocols have their own benefits and drawbacks, to highlight the characteristics of each energy harvesting MAC protocol, we need to have a precise comparison of the existing energy harvesting MAC protocols.

For this reason, we provide a comparison of existing energy harvesting MAC protocols according to the categorization presented in Section 3. Then, for each channel access category, we consider two sets of parameters, which are not absolute values. The first group of parameters is related to the common features (extracted from the existing literature) of the energy harvesting proposed MAC protocols regardless of their channel access categories, and the second set of parameters is defined based on the specific requirements of each channel access category. Since the parameters from the first set are common among all four categories, we list them at this point, and the specific parameters of each category will be explained within their related category. The common set of parameters include the type of the energy harvester, whether the MAC protocol mechanism is energy-efficient and is designed based on ENO condition, and probabilistic approach. Energy efficiency is an important parameter in the context of energy-aware MAC protocols since their goal is to reduce energy consumption to adapt to energy harvesting. Hence, the listed MAC protocols in Section 3 do not introduce energy efficiency at all, but the MAC protocols in Section 6 try to modify and enhance the Section 3 protocols in a way to increase the efficiency in terms of energy. The ENO condition has not been satisfied in any energy harvesting MAC protocol included in this survey, and it is more like a benchmark for future works. It is worth mentioning that the probabilistic approach in this context means that the MAC protocol makes decisions based on the previously gathered information and refers to the techniques that the MAC mechanisms adopt to manage the available energy of the nodes (estimation of the energy level and dynamic change of the MAC parameters based on the network conditions). Further parameters include whether the MAC mechanisms adapt to the variable amount of available energy or not and prioritize the frame transmission or not. For each energy harvesting MAC protocol, the specific energy management techniques which are deployed in the mechanism, and the type of IoT application that is supported, are highlighted.

In this section, the mechanisms of existing energy harvesting MAC protocols in the literature are explained, then, some of the advantages and disadvantages of these protocols are highlighted. Finally, some of their modifications and enhancements are listed.

### 6.1. Random Access

In the random access category, the increasing trend of collision rate can be alleviated by balancing the trade-off between collision rate and parameters such as idle listening, overhead reduction, load balancing, and QoS support.

#### 6.1.1. Carrier Sensing-Based Energy Harvesting MAC Protocols

The collision management techniques used to reduce the energy consumption in carrier sensing-based MAC are divided into two main approaches known as channel prioritization and forced to leave the contention.

The first approach is channel prioritization in which the network energy consumption is reduced by adjusting the wake-up duration to the energy level of an individual node and prioritizing the frame transmissions based on their contents. One of the earliest energy harvesting MAC protocols, which is mostly known as the reference mechanism of this approach, is Radio Frequency MAC protocol (RF-MAC) [145]. This protocol intends to balance the trade-off between data frame and energy transmissions at the same frequency band. Also since the data frame transmission is based on the random contention window values, nodes with a high level of energy do not access the channel more than those nodes with a low energy level. Although this protocol reduces some amount of energy consumption of the network, it faces a few challenges, such as long transmission delay due to the random back-off and harvesting procedures and lack of providing the QoS requirements. Since this protocol is designed based on the CSMA method and does not provide time synchronization, optimizing the output power of high-frequency signals with different phases, is another challenge for this protocol.

One solution for the shortcomings of RF-MAC is presented in [146], where an algorithm allows an on demand energy harvesting within contention (back-off) period, to reduce the delay. However, in networks with high traffic and frequent energy harvesting procedure, this MAC protocol still suffers from unpredictable and long transmission delays.

In the second approach, active nodes are randomly forced to leave the contention and go to sleep mode. Consequently some amount of the energy budget of the network is saved. One of the earliest energy harvesting MAC protocols that adopted this approach is Energy-Level MAC protocol (EL-MAC) [147]. This protocol divides the nodes into primary (higher energy) and secondary nodes (lower energy). First, it gives the access channel to the secondary nodes with a lower level of energy and forces the primary nodes to go to sleep mode. For this reason, this protocol conserves energy by reducing the contention level and providing channel prioritization. However, since nodes with a high level of energy are forced to harvest energy while they stay in sleep mode, they lose the opportunity to contend.

Although the second approach improves the trade-off between collision rate and idle listening more than the first approach, neither considers load balancing or overhead reduction as energy-related parameters.

#### 6.1.2. Blind Access-Based Energy Harvesting MAC Protocols

A mathematical model of an energy harvesting MAC protocol, which is designed based on an integration of the RF harvester with the Slotted ALOHA mechanism, is proposed in [148]. According to this mechanism, each node includes a data frame buffer with a single frame capacity and an energy frame buffer with the capacity to transmit a certain amount of energy frames. Data frame transmission for each node requires a specific amount of energy frames. Since in this mechanism, the data frame and energy buffer highly interact with each other, it is able to model only small-sized networks. Also, in this mechanism, long transmission delay reduces the number of arriving energy frames, which increases the data transmission failure.

To reduce the transmission failure in small-size networks, the authors in [149] introduced a Hybrid AP (HAP), which controls frame synchronization and channel access prioritization. An enhancement of harvest-then-transmit protocol [150], defines a dynamic energy harvesting duration for each node to support dense networks, reduce the implementation complexity, and frame transmission overhead. However, delay due to long idle listening duration remains an open issue, and due to the lack of any exact performance measurements, it has not been deployed in wireless environments.

#### 6.1.3. Analytical Discussion of Random Access Category

The aforementioned random access-based energy harvesting MAC protocols are just a few protocols considered as the reference protocols belonging to each approach. Other random access-based energy harvesting MAC protocols are listed in Table 7. As described in this section, most of the protocols focus on collision management techniques, which may increase the idle listening duration, and thus increase the network energy consumption. Moreover [146,151], intend to improve RF-MAC in terms of energy efficiency by applying various energy management techniques such as adaptive contention window algorithm, energy-aware RTS/CTS. However, the ENO concept, which adjusts the performance of the protocol to the chaotic behavior of the harvested energy, is taken into consideration only in [146,152]. In contrast to the carrier sensing approaches [149,150], approaches reduce the overhead. However, in both subcategories, the missing energy-related parameter among the proposed MAC protocols is load balancing. The carrier sensing-based approaches were mainly designed based on in-band RF technique, whereas blind access approaches are considered out-of-band RF technique. A few of the listed MAC protocols in Table 7 are evaluated based on real measurements, while the rest are evaluated through analytical models and simulations. Among all the protocols, QAEE-MAC [153], QPPD-MAC [154] and DeepSleep MAC [155] protocols fulfill most of the energy conservation parameters, which are highlighted in this table.

### 6.2. Scheduled Access

In the scheduled access category, node synchronization is one of the main issues that have an impact on the energy consumption of the network [156]. Other energy-related parameters that need to be taken into account for this category include end-to-end delay, resource allocation, overhead, and interference.

#### 6.2.1. Channelization-Based Energy Harvesting MAC Protocols

Predefined frame assignment avoids collision and makes the idle listening duration unnecessary. However, fixed assignment methods require global time synchronization, which increases the overhead and thus increases the network energy consumption. Among all methods of this category, TDMA tackles overhead issues more efficiently.

One of the reference protocols in this subcategory is the Energy Harvesting TDMA (EH-TDMA) [156], which assigns predefined frames to the nodes for data transmissions, and controls these transmissions by sending a small frame known as a ping message. In the EH-TDMA MAC, all nodes have the responsibility to harvest the energy whenever possible. EH-TDMA is designed for single-hop scenarios, and improves channel utilization, interference reduction, and is evaluated based on the simulations on the three different radio platforms. Since the EH-TDMA mechanism does not provide an energy management method to estimate the future energy level based on the existing level of energy, it cannot manage the energy budget of dense networks and intermediate nodes efficiently and may not be practical for them.

The network energy consumption can be managed through NOMA-based approaches. In [157], the protocol dynamically defines the duration of energy transfer based on the number of active nodes in the network, to optimize resource allocation. Since this modification does not consider the future energy level of each node, it faces a long delay due to global time synchronization. Another NOMA-based modification of EH-TDMA MAC protocol is proposed in [158], where the transmission power is reduced to support dense networks. Although these protocols manage network energy consumption, they suffer from hardware (especially receiver) and computational complexity.

#### 6.2.2. Controlled Access-Based Energy Harvesting MAC Protocols

The proposed MAC protocols in this subcategory are primarily designed based on the polling method rather than the token passing method.

One of the earliest energy harvesting MAC protocols in this subcategory is a Probabilistic Polling mechanism (PP-MAC) [159], which is evaluated based on real measurements. This protocol broadcasts a defined contention probability through the network. The coordinator adjusts this value based on the network conditions, such as the energy harvesting rate of each node and the size of the network, which can be changed dynamically. Although PP-MAC protocol provides fairness in the network, it changes to network dynamics very slowly, which increases the transmission delay and cannot support scenarios with intermediates nodes, interference, and hidden terminal issue.

One solution to address these shortcomings is the method Estimated Number of Active Neighbors (ENAN) [160], which dynamically adjusts the contention probability to the energy harvesting rate, thus reducing the collision rate and the number of polling frames simultaneously. In contrast to polling-based mechanisms, which face long delays due to synchronization, token-based mechanisms reduce it by eliminating the synchronization from the channel management procedure.

Although the channelization-based protocols require global synchronization, control access-based mechanisms such as Probabilistic Polling and Token passing require local synchronization, and thus they can reduce the transmission delay. However, two crucial energy-related parameters, overhead and high channel utilization, are missed in the scheduled access-based energy harvesting MAC protocols.

#### 6.2.3. Analytical Discussion on Scheduled Access

The aforementioned scheduled access-based energy harvesting MAC protocols are just a few protocols that can be considered as the reference protocols belong to each approach. Other scheduled access-based energy harvesting MAC protocols are listed in Table 8. As described in the table, most of the proposed energy harvesting MAC protocols belong to this category, are equipped with RF energy harvesters to prolong the lifetime of the wireless devices. However, these protocols do not consider the ENO concept and cannot reduce the energy consumption of the communication process. Also, they apply various energy management techniques such as power allocation and grouping strategy to reduce the energy consumption of the network. Some of the presented MAC protocols in Table 8 are evaluated based on the empirical measurements whereas, others perform simulations. Among all these protocols, EH-TDMA MAC [156] and EH-MAC [160] satisfy more features towards the MAC layer energy conservation.

### 6.3. Hybrid Access

In hybrid access category, due to the combination of random and scheduled access categories, the main issues that impact the energy consumption of the network are collision rate and node synchronization. The relevant parameters to collision management are overhead, load balancing, QoS requirements, and grouping strategies. At the same time, the relevant parameter to node synchronization is resource allocation. In this subsection, we list such hybrid schemes that combine random and scheduled access.

#### 6.3.1. Combination of Blind Access and Channelization Subcategories

One of the earliest versions of the Dynamic Frame ALOHA (DFA) MAC protocol with energy harvesting is introduced in [161]. In this protocol, a coordinator node initializes a query command, which synchronizes the nodes and schedules the order of channel access for each node. Then each node, which is equipped with an energy storage device and a harvester, is allowed to start the data transmission procedure at an inventory round according to the DFA mechanism. The authors highlighted that the frame size needs to be dynamically adjusted to the energy harvesting rate and energy level of the nodes. Moreover, the required energy of each frame depends on the reception of a query message, acknowledgment, or transmission of a data frame. This protocol is evaluated based on a mathematical model and intends to provide a balance between the complexity level of implementation and the size of the network. However, this protocol suffers from high level of energy consumption, due to the node synchronization and the increasing trend of collision rate under high traffic load.

To alleviate energy wastage due to node synchronization, an ALOHA-based approach is presented in [162] which defines a grouping procedure. In this protocol, instead of individual node synchronization, the synchronization is defined for each group of nodes, and thus the energy wastage due to this issue is decreased. Another enhancement of the DFA MAC protocol is presented in [163]. Collision rate reduction is achieved by combining the NOMA method with the SA method, where the nodes start transmission based on different predetermined power transmissions. Nevertheless, addressing some energy-related parameters such as long transmission delay, overhead reduction, load balancing and QoS requirements still remain unsolved.

#### 6.3.2. Combination of the Carrier Sensing and Channelization Subcategories

An early energy harvesting MAC protocol based on the combination of CSMA/CA mechanism and TDMA method is an adaptive energy harvesting MAC protocol, which is proposed in [164]. This protocol divides the frame into four different parts. In the first period, the controller node sends a notification to all the nodes to prepare them for the energy harvesting period. In the second period, only nodes that contain a frame can start harvesting energy. Then in the third period, these nodes start to contend to access the channel based on the CSMA/CA mechanism. In this phase, according to the energy level of each node, a certain contending probability is assigned to them (nodes with a lower level of energy have a higher contending probability). In the end, the successful nodes transmit data frames according to the TDMA method. This protocol is evaluated based on a mathematical model, which reduces the transmission delay, number of collisions and optimizes the energy harvesting period. Hence, in this protocol, energy harvesting is fulfilled in an out-of-band manner, no interference occurs between energy and data frame transmissions. However, in the case of in-band energy transfer, the MAC protocol needs to be designed in a way to avoid energy and data frame interference.

One solution to this issue is presented in [165], where an interference cancellation technique is applied to make sure that all the nodes have sufficient energy to operate, and utilizes an adaptive sleep duration management to provide node synchronization and reduce the collision rate. Another method to prevent the energy and data frame interference is the clustering approach, which is presented in [166]. For this reason, the active time of the cluster heads is reduced to an optimal value. However, providing individual charging time for each node, or using a clustering approach, make these protocol implementation complex for the coordinator and they cannot meet the QoS requirements.

#### 6.3.3. Combination of Carrier Sensing and Controlled Access Subcategories

An example of this technique is Human Energy Harvesting for WBANs (HEH-BMAC) [47] protocol, which is evaluated based on extensive simulations and can be applied to realistic networks. The main target of this protocol is to prioritize channel access based on the data traffic type. Hence, the data traffic load is divided into two types, data with high priority and data with normal priority. The ID-Polling MAC protocol, which provides contention-free channel access, is used for high priority transmissions, and CSMA is used for nodes with regular priority transmissions. The HEH-BMAC dynamically schedules the duration of each procedure based on the energy level of nodes. According to the authors, this protocol adapts to the changes in network size and energy harvesting rate and reduces the transmission delay. However, mathematical evaluation of the proposed MAC protocol, QoS of the network, and introducing a smart energy-efficient algorithm have been considered as the future work.

An early enhancement of this protocol [167] intends to reduce the collisions and move towards a smart energy-efficient approach. For this reason, it combines a wake-up/sleep scheduling approach and ENAN mechanism (where the coordinator node frequently updates the polling probability), to reduce the number of missed poll frames, and thus decreases the number of re-transmissions. However, transmission delay has not been evaluated in this work and is considered as an open issue. Similar to HEH-BMAC, this protocol has not been designed for ENO conditions, and supporting QoS and load balancing as energy-related parameters remain open challenges.

#### 6.3.4. Switching from Random Access to Scheduled Access Categories

The Receiver-Initiated Harvesting-aware MAC (RIH-MAC) protocol [168] adopts a fixed assignment reservation method in direct communications, where a controller schedules the frame transmission procedure according to the information of nodes. In contrast, in the absence of the controller node (ad hoc), it operates based on the CSMA/CA mechanism. In both cases, the transmission procedure is started when the receiver has harvested enough energy to send the Ready to Receive (RTR) message to the nodes. Then, these nodes are only allowed to send their data frames after the reception of the RTR message. The RIH protocol adapts to the size of the network and reduces the number of collisions. Hence, it saves some amount of energy budget of the network. However, it still suffers from the hidden terminal issue and long transmission delay.

#### 6.3.5. Duty-Cycled-Based Energy Harvesting MAC Protocols

The reference MAC protocols based on this approach are two dynamic wake-up/sleep scheduling protocols known as Duty-cycle Scheduling based on Residual energy (DSR) and Duty-cycle Scheduling based on Prospective increase in residual energy (DSP) [169]. The DSR protocol reduces the delay duration due to the sleep mode, while the DSP protocol adjusts the duration of wake-up/sleep mode to the estimation of the increasing amount of the residual energy based on the available harvested energy (it reduces the sleep latency). These two protocols are evaluated based on extensive simulations utilizing Network Simulator 2 (ns-2).

One of the modifications of these two protocols, which intends to reduce the energy wastage during the idle listening mode, is proposed in [170]. ODMAC protocol adjusts the wake-up/sleep duration to the current residual energy of each node and saves the energy budget of the network. However, it faces two unsolved issues, it does not apply any mechanism to control frame re-transmissions and it suffers from the hidden node problem. To address the energy wastage due to the re-transmission an exponential decision MAC is introduced in [171], where the wake-up/sleep scheduling is defined based on the future residual energy of each node (intelligent scheduling). Although this protocol outperforms ODMAC in terms of energy consumption, it does not point to the hidden node issue and its computational complexity costs a longer transmission delay. A more recent enhancement of DSR and DSP protocols is proposed in [172], where not only wake-up/sleep scheduling, but also the contention window, is adjusted to the amount of harvested energy and energy harvesting rate. This protocol outperforms ODMAC and ERI-MAC in terms of network energy consumption by reducing the level of contention. However, the performance of this protocol has not been evaluated under dense networks condition.

A realistic evaluation is performed in [173] based on the Synchronized Wake-up interval MAC (SyWiM) protocol. This protocol benefits from a solar cell and is designed based on the Receiver Initiated Cycled Receiver (RICER) MAC protocol. It intends to improve the QoS, reduce the delay, load balancing, and total energy consumption of the network by considering the ENO condition. According to this protocol, nodes stay in a harvesting or a non-harvesting period. The energy consumption of the non-harvesting period is reduced by dynamic adaptation of the wake-up/sleep interval to the residual energy of the node, and in the harvesting period, this interval adapts to the harvested energy. The SyWiM protocol is validated on a real implementation; however, by increasing the number of nodes, the performance of the network may be affected. In duty-cycled-based energy harvesting MAC protocols, balancing the trade-off between energy conservation and transmission delay still is a challenge. Moreover, introducing new methods of wake-up/sleep adjustment to the features of the battery, such as available energy in a battery, its capacity of loss, and charging profile, remain open issues.

There is two main differences between duty-cycled-based energy harvesting MAC protocols and other hybrid-based ones. First of all, thanks to the duty-cycle techniques, these protocols balance the trade-off between collision rate and idle listening duration. The second difference relates to their capability to support QoS.

#### 6.3.6. Analytical Discussion on Hybrid Access Category

The aforementioned hybrid access-based energy harvesting MAC protocols are just a few that can be considered as the fundamental protocols in this category. Other proposed protocols, which are listed in Table 9, mainly reduce the energy consumption of the network by applying energy management techniques such as adaptive wake-up/sleep scheduling, probabilistic contention, and collision management. However, they still do not consider the ENO concept. Due to the combination of the random and the scheduled access, the deployed energy harvesting techniques in the hybrid access protocols can be adapted to a random access approach or a scheduled one. Also, these protocols support a wide variety of application types from healthcare applications (HEH-BMAC [47]) to M2M applications (DFSA and EH-RDFSA [174,175]). Among all these protocols, the AH-MAC [166] and SyWiM [173] are two protocols, which support most of the listed parameters and have been evaluated through a real test-bed.

### 6.4. Cross-Layer

The energy-related parameters in this category are taken from the lower layer (power transmission) or upper layer (optimal path selection) of the MAC layer in the IoT protocol stack, whose information helps the MAC layer to make an optimal decision. Also, other parameters, such as grouping strategy and the number of connected layers, can express useful information regarding the complexity of the implementation of the proposed approach. In the following subsection, existing literature on other protocol layers that assist the MAC in improving the energy efficiency are explained.

#### 6.4.1. Interaction between the Physical Layer and MAC Layer

The presented MAC protocol in [176] intends to combine information of the physical layer with the MAC layer performance. It dynamically adjusts the duration of the wake-up/sleep scheduling and the transmission power to the harvested energy level and the quality of the link. These adjustments are jointly made based on the Exponentially Weighted Moving-Average (EWMA) algorithm and the last Received Signal Strength Indicator (RSSI). This cross-layer mechanism deploys a Transmitter Initiated Cycled Receiver MAC (TICER) protocol to manage the contention level of the channel. The proposed protocol is implemented in the PowWow [177] platform, with a solar cell and a super capacitor to save the harvested energy. This protocol is evaluated based on a realistic network and provides energy efficiency in a real IoT system.

#### 6.4.2. Interaction between the MAC Layer and Network Layer

The Opportunistic Wake-Up MAC (OPWUM) [178] protocol which belongs to this subcategory, benefits from the information of the routing table and hence, reduces the energy consumption of the transmissions. This protocol operates based on the CSMA/CA mechanism and selects the receiver node among several receivers in an opportunistic manner (opportunistic forwarding). The transmission procedure is started when a node sends the RTS frame to all the potential receivers. Then, the potential receivers adjust their back-off timers to the state of a specific metric, which is defined according to the application requirements. For instance, one metric can be the level of residual energy. In this case, the receiver nodes with a higher level of residual energy have a higher priority to respond to the node. The OPWUM protocol is evaluated based on a mathematical model and then is implemented in GreenCastalia. This protocol reduces the level of contention and total energy consumption of the network. However, other parameters, such as transmission delay and network size, are not considered.

#### 6.4.3. Interaction between the Physical, MAC, and Network Layers

The cross-layer approach, which is presented in [67], aims to balance the trade-off between energy consumption and the duration of the wake-up/sleep scheduling approach. This protocol deploys a geographic routing protocol known as Two-Phase Geographic Greedy Forwarding (TPGFPlus) at the network layer, and it adjusts the transmission power to the residual energy level at the physical layer. The MAC layer of this method operates based on the Connected K-Neighbourhood (CKN) sleep scheduling algorithm. This algorithm periodically adjusts the wake-up/sleep duration to the residual energy level of the nodes. Each node decides to stay in sleep mode or active mode based on the collected information from the two-hop neighbor locations, energy harvesting rate, residual energy, and the energy consumption of the network. This dynamic decision reduces the number of collision rate; however, different energy-related parameters such as load balancing, overhead reduction are not addressed in this protocol.

The Cross-Layer MAC Energy Harvesting Sensor Node (CL_EHSN) [179] is another protocol that belongs to this group. In the CL_EHSN protocol, first of all, a path is established between two nodes with the help of routing protocols. Then, the MAC protocol based on this information decides about the next-hop nodes. The fundamental of the MAC protocol is based on the four-way handshaking CSMA/CA mechanism and is responsible for determining the charging and active duration of the node. According to the residual energy level of each node, the node decides whether to start the transmission procedure or to start harvesting energy. For this purpose, the node sets its antenna at Transmit/Receive (Tx/Rx) mode and then switches its antenna to energy harvesting mode for the rest of the time. The CL_EHSN provides a flexible and energy-efficient discovery path method. Also, in the case of the dense network, the CL_EHSN outperforms conventional protocols in terms of long transmission delay issues.

#### 6.4.4. Analytical Discussion on Cross-Layer Category

Table 10 summarises the characteristics of energy harvesting MAC protocols based on the cross-layer approach. The main target of these protocols is to satisfy the ENO concept and then evaluates them based on a real implementation. These protocols mostly benefit from the adaptive wake-up/sleep scheduling technique. Among all the mentioned protocols in Table 10, the CL_EHSN MAC addresses most of the parameters that need to be considered towards enabling energy harvesting techniques in wireless communication systems. Since hybrid access and cross-layer approaches are designed based on the combination and integration of different techniques and layers of the network model, these approaches may increase the computational process of the protocol which requires further optimizations. Although the Low Energy Self-Organizing Protocol (LESOP-MAC) [54] does not consider an energy harvesting technique, it makes the integration of the energy harvesting techniques possible.

## 7. Open Issues and Research Challenges for Energy Harvesting MAC Protocols within IoT Systems

According to Table 5, energy harvesters provide a wide range of output power from 3 nW to 100 mW, which is produced by an AESC and a solar cell, respectively. Moreover, energy models, which are explained in Section 4 show that the power consumption of wireless communication technologies is much higher than the scavenged energy through the well-known energy harvesters listed in Table 5. For instance, according to Table 6, a radio frequency antenna which provides a power density of at most 0.3 mW/${\mathrm{cm}}^{2}$, cannot support IEEE 802.11ah frame transmission in different applications such as agricultural monitoring (mean current consumption 0.12 mA) or smart metering (mean current consumption 0.045 mA). Since IoT systems include a large number of devices with limited size, the energy insufficiency which lies between the energy harvester device and wireless communication device power consumption becomes worse.

**Figure 6:**
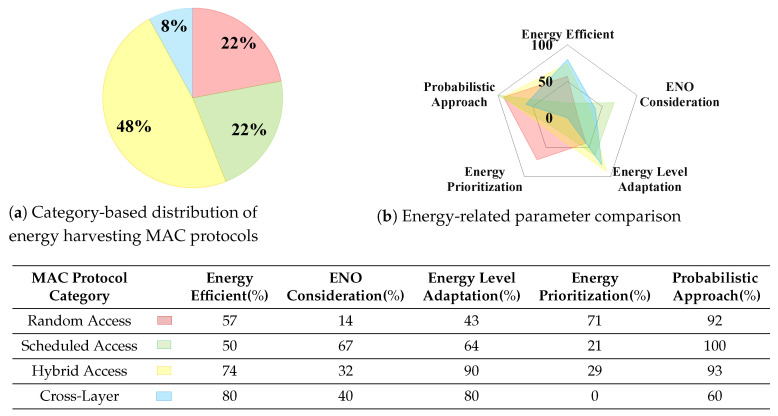
Category-based comparison of energy-related parameters in energy harvesting MAC protocols.

**Figure 7:**
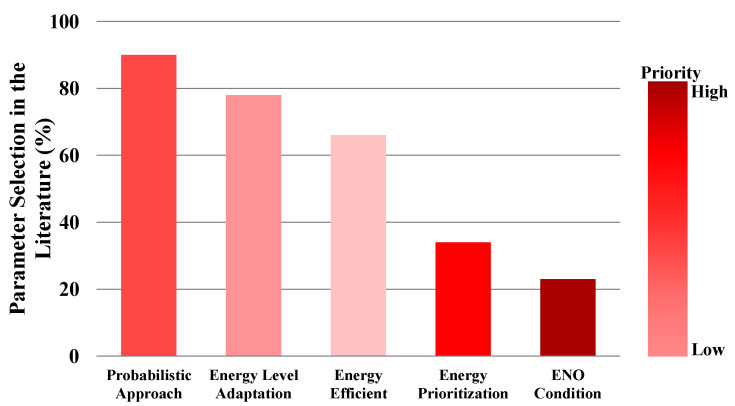
General comparison of energy-related parameters in energy harvesting MAC protocols.

In IEEE 802.11, around 80% of the total energy budget of the whole network is wasted by the MAC layer anormalies (collision frame, idle listening, overhearing, overhead) [180]. Thus, as mentioned in Section 6, to make the energy harvesters applicable for integrating with the existing wireless communication technologies and alleviate the energy wastage of MAC layer anormalies, different enhancements or modifications of the currentenergy-efficient MAC approaches in the literature are proposed. A comparison of the common feature of the energy harvesting MAC mechanisms, which are explained in Section 6 is summarized in Figure 7. From this figure, it can be perceived that among the selected energy harvesting MAC protocols, cross-layer mechanisms propose the most energy-efficient methods by considering energy level adaptation. In the random access category, 57% of the protocols are energy-efficient, whereas in the hybrid access category, this value increases to 74%, and 90% of these hybrid protocols adapt to the energy level of the node. In contrast to the random and the scheduled access categories that allocated 14% and 67% of the protocols to ENO condition, in the hybrid access category 32% of the protocols consider this condition. Also, according to the analysis results (cf. Figure 7), 23% of the existing energy harvesting MAC protocols in the literature consider the ENO condition, which is the key parameter in IoT systems equipped with energy harvesters. To address energy efficiency in energy harvesting MAC protocol designs, 66% of related works have applied different energy optimization methods and energy management techniques. Although 90% of the MAC protocol designs have adopted a probabilistic approach, 73% of them schedule the node transmissions based on the available level of the energy in the node, and only 34% prioritize the transmission of the nodes with a lower level of energy. Figure 6 and Figure 7 convey that, since only a reduced set of features have been considered in the design of the proposed energy harvesting MAC protocols, there still exists room for improvement in designing energy harvesting MAC protocols. Also, they show that reflecting all the essential considerations to enable energy harvesting techniques at the MAC layer remains a challenging issue, specifically in hybrid access and cross-layer categories that can optimize the performance of MAC layer operations.

In the first part of this section, we will list the energy wastage at each level of IoT systems, and then we will focus on the role of MAC layer operations regarding energy wastage. In the second part of this section, we will expand the existing challenges that can lead to future research in these topics.

### 7.1. Essential Considerations to Make MAC Protocols Applicable for Energy Harvesting Techniques

To enable energy harvesting techniques at the MAC layer within the communication level of IoT systems, different challenging issues need to be considered. The most relevant considerations are described next.

#### 7.1.1. Energy Optimization at Different Levels of IoT Systems Architecture

IoT systems are defined by academia and industry based on three levels known as, management level, communication level, and end device level. Our focus in this paper is the optimization at the communication level. The most relevant approaches in the literature to achieve this goal are described next.

1. Optimization at management level
  The management level in IoT systems architecture refers to data centers and cloud computing. Estimations show that around 1% of the worldwide energy budget is spent in data centers [181], thus different works in the literature [182,183,184] have been oriented towards optimizing the energy consumption at the management level. Along with these methods, in LoRa and Sigfox proprietary standards duty-cycle regulations, which are defined at the management level, can manage the energy consumption of the network by setting the duty-cycle at 0.1% and 1.0% duty cycle per day based on the channel (in Europe). However, since the MAC layer operations do not have a direct connection with this level, the energy optimization at the management level is out of the scope of our study.
2. Optimization at communication level
  At the communication level of the IoT system architecture, the energy consumption of the networks (including those equipped with energy harvesters) can be optimized through different approaches. One approach is the cross-layer framework design [185], which may exclude network and transport layers from the IoT protocol stack to avoid operations of these two layers, or use the information from the network and physical layers to enhance the performance of the MAC layer operations. However, merging different layers of IoT protocol stack is a challenging issue and requires some predefined standardization [185].
  The second approach refers to the optimized energy consumption of the network by selecting the best placement of the gateways or APs [186]. In this approach, each gateway can create a cluster to address specific constraints such as connectivity coverage range, shortest path selection, transmission power, and resource allocation. This grouping strategy could help the MAC layer to optimize the transmission scheduling according to the requirements of each group. Although grouping strategy can be a challenging issue in terms of prediction models, it may reduce the number of required sensors to fulfill a measurement.
  The third approach refers to various PSMs that are deployed in different wireless communication technologies. These methods mainly send the node to the sleep mode state to save energy, however changing the sleep mode to wake-up mode several times has some drawbacks such as long delay. For this reason, there has been many studies in the literature to balance the trade-off between PSMs and other KPIs of the network [155], however, this issue still requires more attention.
  The fourth approach refers to the application layer IoT protocols like Constrained Application Protocol (CoAP) and Message Queue Telemetry Transport (MQTT). Since these protocols are considered lightweight protocols with small header sizes, they reduce the energy consumption of the network. However, due to their lack of direct impact on the MAC layer, a study on them is out of the scope of this study.
3. Optimization at sensing and perception level
  This level mainly includes sensors, actuators and edge devices, which interact with the environment. For this reason, the optimization methods are designed for processor, and wireless transmitters. For instance, one approach is WUR, which is designed based on the communication range and other characteristics of the networks, however it suffers from wake-up beacon collisions. For this reason, designing an energy-efficient WUR is a challenging issue, which has been addressed in literature [24] and but still requires more research.
  The other approach refers to optimizing the central unit processing-memory related power consumption in IoT end devices. This approach is more challenging for non-real time applications, which require a prediction of the deadline, arrival time, and workload of each task beforehand. For instance [187] proposes a dynamic voltage and frequency scaling method, which can adapt to the nature of the non-real time applications by using a machine learning algorithm. The authors demonstrated that their proposed algorithm reduces 42% of the Central Processing Unit-memory (CPU-memory) related energy consumption.
  Besides, in the CL_EHSN cross-layer mechanism [179], which is explained in Section 6, the processor-memory related power consumption can be reduced through the energy-aware MAC, which makes the decisions based on the status of the battery, and estimating the charging time.
  The implementation complexity of the MAC protocol, or its algorithms, is another issue that consumes some parts of the network energy consumption. Since complex algorithms require more computation time, they may consume more energy. Nevertheless, the optimization methods complexity should be suitable to the requirements of each level. For instance, channel access complexity should be managed by the central units or APs and should not move to the sensing level.

#### 7.1.2. Energy Optimization for Different MAC Anormalies

According to Section 6, IoT systems benefit from different channel access mechanisms. However, each mechanism has its own drawbacks. For instance, in random-based access mechanisms, energy is wasted due to the re-transmissions, and extra overhead exchanging frames. In scheduled-based access mechanisms, the energy wastage is caused due to the node synchronization or idle slots. The most relevant reasons why MAC protocols in wireless communication devices waste energy are detailed next.

1. Collision frame
  In Random-based access mechanisms, collision frame occurs when two or more nodes try to send data frames over the shared channel simultaneously. The collision causes data frame discarding, which requires a re-transmission. Thus, due to the frame re-transmissions, the energy consumption increases. The authors in [188] showed that in the DFSA mechanism the amount of wasted energy due to the collisions varies from 0.1 to 1000 mJ for different network densities. To alleviate this issue, different approaches such as NOMA [158,163] have been proposed, however, still more researches are required to reduce collision rate and conserve energy.
2. Idle listening
  Although the time listening to the shared medium and waiting to start the frame transmission controls the collision rate, it causes extra energy consumption specifically in random access and hybrid access categories. According to the literature [189], between 80–90% of the energy wastage of data transmission procedure in the distributed mechanisms is related to the idle listening duration. The authors in [155,171,188,190] showed that depending on the network density and mechanism’s constraints, energy wastage during idle listening can be varied from 1 mJ to 1 J. Although to balance the trade-off between the idle listening duration and collision rate, different researches have been done [191], finding an optimal value for this duration still remains an open challenge. This issue in the context of duty-cycled mechanisms has a different definition. Based on the data frame transmission in duty-cycled mechanisms, some amount of energy can be wasted, due to idle listening mode. This means a receiver node stays in idle listening mode and waits for receiving a data frame, while no data frame has been sent by the senders.
3. Overhead
  Although control frames do not contain any data, they are necessary for communication management. In random access and controlled access approaches, different QoS requirements, control messages, and long headers within data frames consume extra energy during the transmission procedure. The authors in [192] showed that the energy consumption of the overhead control packets in IEEE 802.15.4 varies from 0.1 to 2 J based on the network density. For these reasons, the overhead reduction issue has been attracting researcher’s attention in recent years, and some solutions such as frame concatenating (superframes) have been proposed to alleviate the overhead issue.
4. Overhearing
  In the case of a dense network, overhearing (i.e., receiving data frames from other transmissions) intensifies and increases the energy consumption of the network. The authors in [180] proposed a decomposition of energy consumption in IEEE 802.11. Since overhearing depends on the size of the network, it changes from 0.01 to 0.11 mJ. According to Section 6 this issue has received less attention rather than collision rate and overhead issues.
5. Hidden terminal
  In random-based access mechanisms, a network may waste energy if two nodes start a transmission at about the same time, but are out of range (hidden) of each other. Based on the number of the hidden nodes, the amount of energy wastage may vary from 1 to 4.3 J [193]. This challenging issue can be reduced through the RTS/CTS mechanism, or even by increasing the power transmission. These two solutions cannot be considered as energy-efficient approaches. For this reason, it is necessary to address this issue in a more energy-efficient manner.
6. Node synchronization
  Although node synchronization approach provides a collision-free data transmission, it is considered the main energy wastage of the channelization MAC protocols [194]. The reason for high energy consumption in this approach is that synchronization requires frequent executions at each node with a defined duration [156]. The amount of wasted energy varies depending on the execution frequency and duration of the synchronization at each node.
7. Unused slots stay idle
  Another issue which causes energy wastage in scheduled-based access mechanisms is the predefined frame slots [156]. Since the specific slot (time/frequency /code/power) is allocated to each node, this slot cannot be used by other nodes to transmit data frames. For this reason, nodes without any data frame to send waste the channel resources (e.g., bandwidth) and energy. The amount of energy wastage may vary based on frame size and network density.
8. Fixed slot-frame length
  Scheduled-based access mechanisms having a fixed slot-frame size, may not have a successful energy-efficient transmission depending on the data frames size. For instance, a long data frame may need to be fragmented to be successfully transmitted. Since the fragmented part of the frame needs additional overhead, it wastes the energy of the network. The authors in [195] showed that in the LPWAN, the transmission energy consumption increases with the number of fragmented frames.
9. Adaptive polling interval to the network load
  This issue reflects the impact of the duration between two successive poll phases in the Polling access method. Although, by increasing the time interval between two poll periods, the number of polls is decreased, and thus the network energy consumption is saved, having long time intervals between these two polling phases increases the energy wastage of the network. Hence, although this issue has been addressed in some researches [37,196,197], finding an equilibrium time interval in polling MAC protocols remains a critical issue.
10. Token pass timing
  In MAC protocols which operate based on the token passing method, the controller node allocates a time interval for passing the token frame to all nodes. During this time interval, no data frame transmission is allowed, and thus, this duration causes energy wastage. Moreover, as the number of network nodes is increased, more energy is wasted due to longer time duration.

#### 7.1.3. Application Diversity

As mentioned in Section 1, IoT systems include a wide range of applications from healthcare and smart cities to industrial automation applications. Nevertheless, each of them requires a different level of KPIs. For instance, healthcare applications require communications with a high level of reliability, availability, and low latency in a small service dimension area with a specific data rate value. Whereas agricultural monitoring must guarantee QoS requirements such as high latency communications, wider service dimension area and lower data rate compared to healthcare applications. Also the reliability in these applications is not as crucial as healthcare applications. The available energy harvesting MAC protocols only focus on one or two KPIs and intend to enhance them for certain applications with a specific range of data rates. Moreover, due to the energy shortage, burstiness of traffic, and lack of synchronization in the network, some real applications may operate based on the new concept of intermittent computing [198]. To specifically address the requirement of intermittent computing in terms of energy consumption, it is necessary to balance the trade-off between these KPIs in the design enhancement of an energy harvesting MAC protocol. The compatibility of these MAC protocols with the diversity of the IoT applications still is a challenging issue.

#### 7.1.4. Adaptation to the Network Conditions

The performance of a wireless network mainly depends on the application type, dynamics of the deploying environment, channel, and MAC layer conditions. These aspects specify the required traffic rate, network density and topology (insertion/removal of the network nodes), the propagation loss, node prioritization, noise interference, channel resource utilization, transmission range, within other parameters [166]. To enhance the design of the MAC protocol, all these parameters need to be taken into consideration. For example, in contrast to the scenarios based on star typologies, the network conditions in multi-hop scenarios dynamically change, and thus, some MAC protocol mechanisms such as channelization-based mechanisms are not suitable for them [160,173]. Another example considers mobility management of the node when a node moves from a network domain with some specific conditions, to another one, and force the network to redefine all its parameters. In this case, the MAC protocol needs to adapt its operation to the new network conditions [194].

#### 7.1.5. Energy Prediction Algorithms

According to Table 7, Table 8, Table 9 and Table 10, some of the proposed energy harvesting MAC protocols deploy prediction algorithms such as EWMA, Weather Conditioned Moving Average (WCMA), and Artificial Intelligence (AI) [199] to predict the required amount of energy of the next transmissions, energy harvesting rate, satisfy ENO, and make the unpredictable behavior of the harvested energy sufficient for protocol operations [200]. For instance, Q-learning and self-learning algorithms are deployed in [201,202] respectively, to achieve optimal approach for energy-efficient communications. It is worth mentioning that to make the network perform in an optimal manner, different energy prediction algorithms depend on the various aspects and requirements of the network, need to be applied at all levels of the IoT architecture. Since machine learning or prediction algorithms require a considerable amount of computational resources, implementing them for dense network scenarios can be complex, challenging, and requires further research.

#### 7.1.6. Validation of the Proposed Energy Harvesting MAC Protocols

In Section 6, we observed that most of the proposed MAC protocols are designed based on analytical models. Also, some works are validated through the use of simulations. However, the simulators which are used in these works are mainly custom-based simulators with ideal conditions, hard to compare or reproduce and may not provide reliable results. In contrast to custom-based simulators, packet level simulators such as NS-3, Optimized Network Engineering Tools in C++ (OPNET++), and Objective Modular Network Testbed in C++ (OMNeT++) are capable of modeling the general structure of the networks such as channel condition, physical, MAC and application layers and imitating the real world network conditions. Other than analytical models and simulation approaches, a few of these works extend the validation of their proposed models to the hardware level and test-bed. For instance, the authors in [203] validated their proposed model with the help of the Field Programmable Gate Array (FPGA) platform, solar and RF energy harvesters. Thus a combination of test-beds,(which consider interaction of different levels and components of the real systems to study the behaviour of the whole system), and simulations based on the packet level simulator for validating the energy harvesting MAC protocols, would be a proper method of validation.

#### 7.1.7. Acceptability of the Design of the Proposed Energy Harvesting MAC Protocols

Plenty of novel energy harvesting MAC protocols have been proposed in the literature. However, those protocols closer to already existing standards have more opportunity to be accepted in the industry. For instance, DeepSleep, HE-MAC, and W${}^{2}$P-MAC, protocols are designed based on the IEEE 802.11 standard, and AH-MAC and RF-AASP MAC protocols are designed based on the IEEE 802.15.4 standard. However other novel MAC protocol definitions require changes on the node behaviour, frame exchanges, and complexity of network, which require strong redesigns of existing standards. The reluctance to deploy them may also lie on the backward compatibility of the proposed MAC protocols with existing wireless devices.

### 7.2. Open Research Challenges

Supporting energy harvesting at MAC layer in currently available wireless technologies can be a promising solution to the energy shortage of IoT end devices. Nevertheless, it may raise new challenges and issues at different levels of IoT systems. In this subsection some of the research challenges that still remain as open issues are highlighted.

1. Radio Resource Management (RRM)
  Radio level management plays a critical role in efficiently scheduling and controlling different radio network parameters which have an indirect impact on the MAC layer operations, and can improve some of the MAC anormalies. RRM can be performed statically to schedule parameters such as frequency and channel allocation, antenna heights, and directions, modulation and channel coding, static handover and energy level of the nodes. Our analysis shows, although different proposed MAC protocols in the literature dynamically adjust the power control level to the data rate [204] or directional antenna [205], they do not enable the integration of energy harvesting with the MAC layer of IoT systems. Thus, presenting RRM algorithms to make the operation of the energy harvesting MAC protocols more efficient would be an attractive research direction (e.g., cross layer approaches).
2. Scalability to dense networks
  Since IoT systems may include a large amount of devices, optimal and scalable network deployment for these systems is required. This means that, by expanding the size of the network, the MAC protocol must keep the network performance at a stable level and satisfy the fairness and QoS among the network. One approach, which addresses the requirements of a dense network is the massive Machine-Type Communication (mMTC). This approach focuses on 5G and Beyond 5G (B5G) technologies and intends to provide reliable and efficient communications while reducing the energy consumption of the network [206]. Although this approach provides the possibility of energy harvesting integration with IoT systems, due to the low level of transmit power, energy transfer (which is one of the methods of energy harvesting technologies) in massive communications with long-distance is not efficient. According to the existing literature that has been studied in this paper, although different assumptions have been taken into consideration to simulate the performance of an energy harvesting MAC protocol in a dense network, optimal deployment of the nodes (which is an important issue for nodes equipped with energy harvester) and network scalability, has not been studied at the same time.
3. Heterogeneity among IoT systems
  In IoT applications such as smart cities, devices may operate based on different technologies and protocols with various constraints and characteristics. In contrast to homogeneous networks, heterogeneous networks face new challenges, such as the coexistence of the technologies [207]. The coexistence issue arises when two or more technologies intend to access the same radio frequency band to complete their communication process. Since coexistence can cause interference in communications, heterogeneous networks are more likely to waste energy [208]. Rather than the interference issue, other factors, such as the network structure complexity and lack of proper resource management, may cause energy waste in heterogeneous networks. Although, one solution to conserve the energy in these networks without degrading the network performance, is to define a universal ENO value for the network, it may be a challenging issue and remains an open research direction.
4. Interoperability among IoT devices
  Different applications that belong to an IoT system require various radio interfaces, network structures, and protocols. This diversity among two or more networks which aim to cooperate at different layers is known as interoperablilty and becomes an issue for IoT systems. From MAC layer perspective, since the energy harvesting MAC protocols are responsible for rescheduling the transmissions, timings, and parameters related to the energy level, interoperability may increase the noise, frame loss, resource utilization and energy wastage in the network. Thus, these protocols need to take into account the effect of interoperability, where the standardizing the interactions between networks can lead to new research directions.
5. Towards batteryless networks with intermittent operations
  In batteryless systems, the required energy to keep the device powered, is provided through energy harvesters. However, due to the unpredictable behavior of energy harvesters, the system may face failure. This problem, which is also known as intermittent system problem, faces various challenges at different levels of the network. Since the intermittent networks cannot operate based on the traditional communication protocols, they require modifications and adjustments at communication coordination and scheduling based on the intermittent nature of the network. At this level, a robust MAC protocol and network topology are needed to provide energy-aware protocols and satisfy the requirement of the intermittent systems. Although researchers have been attempting to address these challenges, defining a standardization at each level of this system establishes new research directions.
6. Achieving energy efficiency in fog computing
  This concept refers to moving an enormous amount of computational data operations, management, and storage from data centers and core networks to the communication level of the IoT, where the central units (gateways and APs) are located, to reduce the computational workload of the data centers [209]. Since big data parallel processing which is the key operation of data centers, is a power hungry operation, artificial intelligence approaches are introduced to alleviate the energy consumption of this process. For instance, machine learning algorithms which are known as artificial intelligence approaches use the information of the end devices and then manage the network resources by adapting the MAC layer (e.g., frame size adjustment) of the central units to the behavior of end devices. Thus, artificial intelligence approaches in Fog computing open new research directions in the energy-efficient IoT paradigm.
7. Hybrid approaches for energy harvesters
  Since the energy harvesters that keep IoT systems powered have different harvesting rate, to enable the energy harvesting techniques at the MAC layer, the operations at this layer need to be adjusted to the harvesting rate. The harvesting rate depends on the energy harvester type, environmental conditions and network topology changes. To tackle the dependency of the harvesting rates on the environmental conditions, network topology changes, and to achieve higher output power, some researchers have intended to integrate different energy harvesters. However, the design of hybrid energy harvester may introduce new challenges. These hybrid harvesters may harvest more energy than the system needs which is wasted through the energy leakage in the energy storage devices. To reduce the extra amount of harvested energy, the energy management needs to match the harvested energy with the energy requirements of the IoT applications.
  Alongside this issue, adaptability to different energy harvesting rates may increase the complexity of the MAC protocol, and based on our background study, only a few works deploying hybrid energy harvesters [203]. Thus, hybrid energy harvester deployment and extra harvested energy management are challenges that have not been addressed as much as other issues, which open new directions to researchers.

## 8. Conclusions

The available energy harvesting technologies cannot continuously power up the IoT devices, which are supported by different wireless communication technologies. To make this integration possible, there is the need to optimize the energy consumption on the wireless communication technologies at different IoT layers. Thus, MAC layer operations, which consume most of the energy budget of wireless communication, are the most relevant candidate for applying energy optimization methods and conserving energy. To justify this argument, we provided a thorough review of the MAC layer operations and different MAC optimization techniques, which some of them are employed in the current IoT wireless communication technologies. Then, based on the informative aspects of the MAC layer operations, we developed a unified approach to systematically analyze energy models for each technology. In addition, we extensively studied the available energy harvesting technologies and their constraints. According to this analysis, we showed that, based on the duty-cycle regulation, simple random access mechanism, low energy consumption, and long-range communications, LPWAN technologies are applicable for different IoT use cases. Moreover, since IEEE 802.11ah is specifically designed for IoT systems, it meets various requirements of these systems, such as long-range and low power consumption communications, with higher data rates. In addition, our research on available energy harvesters concluded that technologies like photo-voltaic panels or thermocouples are applicable to these two wireless communication technologies. For these reasons, LPWAN family and IEEE 802.11ah are two of the promising wireless technologies in IoT systems. Also, this paper has described how the available energy harvesting MAC protocols adapt to the integration of energy harvesting in IoT systems, and gave a precise comparison between these MAC protocols based on energy harvesting-related network parameters. These analysis results demonstrated that ENO condition, which is one of the most energy-related parameters for enabling energy harvesting in IoT systems, is only considered by 23% of the reviewed literature set. Furthermore, hybrid access MAC protocols can be one of the optimal approaches for IoT systems equipped with energy harvesters. Their high adaptation to the energy level of the nodes and acceptable network energy consumption reduction favours their presence in 48% of the analyzed literature. Alongside the hybrid access energy harvesting MAC protocols, cross-layer mechanisms show a remarkable energy consumption reduction of the network. However, due to their high computational complexity, they have not reached maturity and have not shown their successful role in the current technologies yet. These results convey that there is still room for improvement in this area. We believe that this survey paper could shed light on enabling the integration of energy harvesting in the IoT concept and guide researchers to explore the adaptation of future MAC layer protocols to energy harvesting techniques in IoT systems.

## Figures and Tables

**Table 1:** Parameters of the energy model equations.

Parameter	Description
$E_{\mathrm{Total}}$	Total energy consumption
$E_{\mathrm{Sl}}$	Energy consumption in sleep mode
$E_{\mathrm{Wu}}$	Energy consumption of wake-up mode
$E_{m}$	Energy consumption during data measurement
$E_{\mathrm{Mcu}}$	Energy consumption of microcontroller processing
$E_{\mathrm{Wut}}$	Energy consumption of wake-up mode for transceiver
$E_{\mathrm{Tx}}$	Energy consumption of transmission mode
$E_{\mathrm{Rx}}$	Energy consumption of reception mode
$E_{\mathrm{Ifs}}$	Energy consumption during the inter-frame space
$E_{\mathrm{Id}}$	Energy consumption of idle mode
$E_{\mathrm{Disconn}}$	Energy consumption of User Equipment(UE) disconnected mode
$T_{\mathrm{Tx}}$	Duration of transmission mode
$T_{\mathrm{Rx}}$	Duration of reception mode
$T_{\mathrm{Id}}$	Duration of idle mode
$T_{\mathrm{Sl}}$	Duration of sleep mode
$T_{\mathrm{Wu}}$	Duration of wake-up mode
$T_{\mathrm{Cd}}$	Duration of cool down mode
$T_{\mathrm{Sw}}$	Duration of switching mode from transmission to reception and vice versa
$P_{\mathrm{Tx}}$	Power consumption of transmission mode
$P_{\mathrm{Rx}}$	Power consumption of reception mode
$P_{\mathrm{Id}}$	Power consumption of idle mode
$P_{\mathrm{Sl}}$	Power consumption of sleep mode
$P_{\mathrm{Wu}}$	Power consumption of wake up mode
$P_{\mathrm{Cd}}$	Power consumption of cool down mode
$P_{\mathrm{Sw}}$	Power consumption of switching mode from transmission to reception and vice versa
*n*	Number of the tag

**Table 2:** Overview of potential technologies for IoT.

Technology	Standard	Coverage	Data Rate	Frequency	MAC	IoT
		Range	(Max)		Protocol	Application
WiFi HaLow	802.11ah	<1.5 km	346 Mbps	Sub-1 GHz	CSMA/CA	Smart City
WiFi-6	802.11ax	<300 m	9.607 Gbps	1–6 GHz	OFDMA	Retail Smart Transportation
WiFi-WUR	802.11ba	<50 m	0.25 Mbps	2.4/5 GHz	CSMA/CA	Smart City Smart Healthcare
LoRa	LoRaWAN	<20 km	<25 kbps	Sub-1 GHz	ALOHA	Smart City
Sigfox	Sigfox	<40 km	0.1 kbps	Sub-1 GHz	RFTDMA	Industrial Automation Smart City
RFID	EPC global Gen2	10 m	423 kbps	850/950 MHz	FSA	Supply Chain
Bluetooth	802.15.1	100 m	3 Mbps	2.4 GHz	TDMA	Smart Transportation Smart Buildings
BLE	802.15.1	100 m	1 Mbps	2.4 GHz	TDMA	Smart Healthcare
ZigBee	802.15.4	<100 m	250 kbps	2.4 GHz	CSMA/CA	Smart Buildings
Z-Wave	Z-Wave	10 m	100 kbps	Sub-1 GHz	CSMA/CA	Smart Buildings
Weightless SIG	Weightless (W/N/P)	5 km	10 Mbps	Sub-1 GHz	SA FTDMA	Smart City
WirelessHART	802.15.4	100 m	250 kbps	2.4 GHz	TDMA	Industrial Automation
NFC	ISO 13157	0.1 m	424 kbps	13.56 MHz	TSCH CDMA/CA	Retail Smart Buildings
THREAD	802.15.4	11 m	250 kbps	2.4 GHz	TSCH	Smart Buildings
ANT+	ANT+ Alliance	100 m	1 Mbps	2.4 GHz	TSCH	Smart Healthcare
NB-IoT	3 GPP	<100 km	250 kbps	Cellular Bands	OFDMA SC-FDMA	Industrial Automation Retail
LTE-M	3 GPP	<100 km	1 Gbps	Cellular Bands	OFDMA	Smart Transportation
EC-GSM	3 GPP	<100 km	2 Mbps	Cellular Bands	TDMA FDMA	Industrial Automation

**Table 3:** Power consumption for IoT technologies (mW).

Wireless Standards	Tx	Rx	Idle	Sleep	Research Methodologies	References
	255	135	1	NA	Analytical Model	Olyaei et al. [115]
					(Simulation OMNeT++)	
IEEE 802.11ah	15.8	6.2	NA	10 × $10^{-3}$	Model Design and Creation	Zheng et al. [80]
					(Matlab Simulation)	
	255	135	NA	1.5	Analytical Model	Raeesi et al. [77]
IEEE 802.11ax	1000	600	300	150	Model Design and Creation (computer simulations)	Yang et al. [84]
IEEE 802.11ba	280	100	50	NA	Model Design and Creation	Hong et al. [89]
	352	154	55	5 × $10^{-3}$	Survey (IEEE Report)	McCormick [85]
LoRa (13 dBm)	18.81	31.65	NA	3.3 × $10^{-3}$	Model Design and Creation	Finnegan et al. [99]
LoRa (13 dBm)	92.4	34.65	NA	0.0033 × $10^{-3}$	(ns3)/Analytical Model	Finnegan et al. [98]
LoRa (7 dBm)	59.4	34.65	NA	0.0033 × $10^{-3}$	Real Measurements	
LoRa (20 dBm)	419.6	44.06	NA	4.32 × $10^{-3}$	Real Measurements	Morin et al. [116]
Sigfox (14 dBm)	214.5	132	1.65	16.5 × $10^{-3}$	Real Measurement	data-sheet [117]
Sigfox (14 dBm)	147	30	NA	4.32 × $10^{-3}$	Real Measurements	Morin et al. [116]
Sigfox Unidirectional	81.6	NA	3.6	48 × $10^{-3}$	Model Design and Creation	Gomez et al. [102]
TXN (14.5 dBm)					Real Measurements	
Sigfox Bidirectional	82.8	55.5	3.6	48 × $10^{-3}$	Model Design and Creation	Gomez et al. [102]
TXN (14.5 dBm)					Real Measurements	
RFID Active Tag	35	28	NA	NA	Model Design and Creation	Namboodiri
RFID Reader	825	125	NA	NA	Simulation	et al. [118,119]
	84	66	NA	NA	Model Design and Creation	Siekkinen et al. [111]
801.15.1 (BLE)					Real Measurements	
	24.11	19.26	4.67	3.24 × $10^{-3}$	Real Measurements	Morin et al. [116]
	90	72	NA	NA	Model Design and Creation	Siekkinen et al. [111]
802.15.4 (ZigBee)					Real Measurements	
	163.74	89.66	40.56	0.165	Experiment	Gray et al. [120]
NB-IoT	852.92	178.34	21.6	0.0108 × $10^{-3}$	Model Design and Creation (Matlab and ns3)	Sultania et al. [105]
	543	90	2.4	0.015	Model Design and Creation	Ratasuk et al. [121]

**Table 4:** Energy source characteristics.

Energy	Form	Life	Cost	Maintenance	Reliability	Scalability	Proper
Source	Factor	Time					Environment
Sun radiation	Medium	High	Medium	Medium	Low	Low	Outdoor
Mechanical	Low	Low/	Medium	High	Medium	High	Indoor/
force		Medium	/High				Outdoor
Dynamic	High	High	High	Low	Low/	Low	Outdoor
fluid					Medium		
Thermoelectric	High	Medium	High	Low	High	High	Industrial area
Acoustic	Low	Low	Medium	High	Medium	Low	Airport/
noise			/High				Railroad
RF	Medium	High	Medium	Low	High	High	Urban area

**Table 5:** Various energy harvesting mechanisms.

Energy	Energy	Power	Limitation	References
Source	Harvester	Density		
Sun	Photo-voltaic	0.15–100 mW/${\mathrm{cm}}^{3}$	Large scale/	Zhou et al. [125]
radiation	panel		Unavailable	
			during night	
Fluorescent	Dye Sensitized	10–100 µW/${\mathrm{cm}}^{3}$	Dependent on	Zhou et al. [125]
light	Solar Cell (DSSC)		the indoor light	Aparicio et al. [126]
Human Body	Piezoelectric	1–2 mW/${\mathrm{cm}}^{2}$	Frangible material	He et al. [10]
Motion		0.2 mW/${\mathrm{cm}}^{2}$		Grag et al. [129]
Vibration	Electromagnetic	0.2 mW/${\mathrm{cm}}^{3}$	Difficult magnet	Grag et al. [129]
	Induction	0.3 mW/${\mathrm{cm}}^{3}$	integration	Zhou et al. [125]
Vibration	Electrostatic	0.021 µW/${\mathrm{mm}}^{3}$	Mechanical	Zhou et al. [125]
	conversion		stability	
Wind force	MicroWindbelt	41.2 mW/${\mathrm{cm}}^{2}$	Unavailable	Laštovička
			in closed area	-Medin [133]
Flowing water	Francis turbine	1 mW/${\mathrm{cm}}^{2}$	Unavailable	He et al. [10]
force			in closed area	
Thermoelectric	Thermocouple	40 µW/${\mathrm{cm}}^{2}$	Low efficiency	He et al. [10]
		50 mW/${\mathrm{cm}}^{2}$		Grag et al. [129]
Acoustic noise	Acoustoelatic Sonic	3 (75 dB) nW/${\mathrm{cm}}^{3}$	Scarce	He et al. [10]
	Crystal (AESC)	960 (100 dB) nW/${\mathrm{cm}}^{3}$	energy source	Yuan et al. [135]
Radio	Wireless Energy	0.1 µW/${\mathrm{cm}}^{2}$	Unavailable	Adila et al. [138]
Frequency	Harvester (WEH)	300 µW/${\mathrm{cm}}^{2}$	in suburban areas	Grag et al. [129]

**Table 6:** Experimental analyses in literature on the suitability of specific energy harvesters with IoT technologies [139,141,142,143,144].

Energy	Energy	IoT	IoT	Power for
Harvester	Harvester Feature	Technology	Use Case	IoT Technologies (%)
Solar Panel	38.5–40.7 ${\mathrm{cm}}^{2}$	LoRa (13 dBm)	Weather Monitoring	98
TEG	4.5 ${}^{\circ }$C (ΔT )	WiFi (IEEE 802.11ah)	Smart Building	10
Human Body (TEG)	3–15 ${}^{\circ }$C (ΔT )	BLE	Smart Healthcare	3.7
Human Body Motion	Located in the shoe with	Zigbee	Smart Building	1.3
(Piezoelectric)	the speed of 6.44 km/h		Smart Healthcare	
Dedicated RF	Tag size	RFID active tag	Industrial Automation	0.3
Harvester	95 mm × 24 mm			

**Table 7:** Comparison of random access energy harvesting MAC protocols.

Protocol	EH Technique	Energy Efficient	ENO Consideration	Adaption Respect to Energy	Prioritization Respect to Energy	Probabilistic Approach	Energy Technique Management	Application Support	Collision Management	Idle Listening	Overhead Reduction	Load Balancing	QoS Support
QAEE	Generic	✓	✗	✗	✓	✓	Adaptive CW	Critical/	✓	✓	✗	✗	✓
MAC	Approach							Urgent Traffic					
QPPD	Solar Cell	✓	✗	✓	✓	✓	Wake-up	Hard to Reach/	✗	✓	✗	✗	✓
MAC							Beacon	Delay Sensitive Application					
RF-MAC	In-Band	✗	✗	✓	✓	✓	Adaptive CW	Generic Application	✗	✓	✗	✗	✗
	RF						Beacon						
REACH	In-Band	✓	✗	✗	✓	✗	Adaptive CW	Real Time	✓	✗	✗	✗	✗
MAC	RF						Application						
HE-MAC	In-Band	✗	✓	✗	✓	✓	EDCA/	M2M Application	✗	✗	✗	✗	✓
	RF						Adaptive CW						
OER-MAC	In-Band	✓	✓	✗	✓	✓	On-demand	Event-driven	✓	✗	✗	✗	✗
	RF						Energy Request	Application					
EL-MAC	Generic	✓	✗	✓	✓	✓	Adaptive CW	Cognitive Radio	✓	✗	✗	✗	✗
	Approach							Networks					
DeepSleep	Ambient	✓	✗	✓	✓	✓	Energy Aware	M2M	✓	✓	✗	✗	✗
	Energy						DeepSleep/	Application					
	Source						Controlled Access						
W${}^{2}$P-MAC	In-Band	✗	✗	✓	✗	✓	ERTS/ ECTS	N/A	✓	✓	✗	✗	✗
	RF												
CEH-MAC	Generic	✓	✗	✓	✓	✓	Cooperation of	Healthcare	✗	✓	✗	✗	✗
	Approach						Harvested	Application
							Energy and Data						
Sakakibara	Out-of-Band	✓	✗	✗	✗	✓	Queuing	N/A	✓	✗	✗	✗	✓
et al.	RF						Mechanism						
Hadzi-Velkov	Out-of-Band	✗	✗	✗	✗	✓	Energy Queue/	Generic	✓	✗	✗	✗	✓
et al.	RF						HAP	Application					
Choi	Out-of-Band	✗	✗	✗	✓	✓	HAP	N/A	✗	✗	✓	✗	✗
et. al	RF												
Harvest-Until	In-Band	✗	✗	✗	✗	✓	HAP	N/A	✓	✗	✓	✗	✗
Access	RF												

**Table 8:** Comparison of scheduled access energy harvesting MAC protocols.

Protocol	EH Technique	Energy Efficient	ENO Consideration	Adaption Respect to Energy	Prioritization Respect to Energy	Probabilistic Approach	Energy Technique Management	Application Support	Delay Reduction	Synchronization Required	Overhead Reduction	High Channel Utilization	Interference Consideration
EH-TDMA	Ambient	✓	✓	✓	✗	✓	Adaptive Wake-up Time	N/A	✗	✓	✗	✓	✓
MAC	Energy Sources						to Energy Level						
TR-EH-TDMA	In-Band	✗	✗	✗	✗	✓	N/A	Heterogeneous	✗	✗	✗	✗	✓
MAC	RF							Networks					
D-TDMA	In-Band	✗	✗	✗	✗	✓	N/A	N/A	✗	✗	✗	✗	✓
MAC	RF												
NOMA-MAC	In-Band	✗	✗	✗	✗	✓	N/A	N/A	✗	✓	✗	✗	✓
	RF												
NOMA-HeTNeT	In-Band	✓	✗	✗	✓	✓	Sub-channel Allocation/	Heterogeneous	✗	✗	✗	✗	✓
MAC	RF						Power Allocation Algorithms	Networks					
NOMA+TDMA	In-Band	✓	✗	✗	✗	✓	Grouping Strategy/	N/A	✗	✓	✗	✗	✓
MAC	RF						SCA Algorithm						
PP-MAC	Solar Cell/	✓	✗	✓	✗	✓	Contention Probability	Monitoring	✓	✓	✓	N/A	✗
	Thermal						Adjustment	Application					
MTPP-MAC	Solar Cell	✓	✗	✓	✗	✓	Grouping	Generic	✓	✗	✗	N/A	✓
							Strategy	Application					
EH-MAC	Ambient	✗	✗	✓	✗	✓	Contention Probability	Event-Driven	✓	✓	✓	N/A	✓
	Energy Sources						Adjustment	Application			
							AIMD/ENAN						
V.B. Mišić	In-Band	✓	✗	✓	✗	✓	Wake-up/Sleep	Generic	✓	✗	✗	✗	✗
et al.	RF							Application					
M.S.I.Khan	In-Band	✓	✗	✓	✗	✓	Queuing Mechanism	Generic	✓	✗	✗	✗	✓
et al.	RF							Application					
EDF-HEAP	Ambient	✗	✗	✓	✓	✓	Earliest Deadline	Monitoring	✗	✗	✓	✗	✗
MAC	Energy Sources						First Polling	Application					
Fair-Polling	Ambient	✗	✗	✓	✓	✓	Contention Probability	Monitoring	✗	✗	✗	✓	✗
MAC	Energy Sources						Adjustment	Application					
Token-MAC	In-Band	✗	✗	✓	✗	✓	N/A	Inventory Management/	✓	✗	✗	✗	✗
	RF							Asset Tracking					

**Table 9:** Comparison of hybrid access energy harvesting MAC protocols.

Protocol	EH Technique	Energy Efficient	ENO Consideration	Adaption Respect to Energy	Prioritization Respect to Energy	Probabilistic Approach	Energy Technique Management	Application Support	Collision Management	Overhead Reduction	Load Balancing	QoS Support	Efficient Resource Allocation	Node Grouping	Packet Fragmentation	Synchronization Required
DFA	Ambient	✗	✗	✓	✗	✓	Backlog	Energy Constrained	✓	✗	✗	✗	✓	✗	✗	✓
	Energy Sources						Estimation Algorithm	IoT Systems								
FA	Generic	✗	✗	✓	✗	✓	Backlog	Generic	✗	✗	✗	✗	✓	✗	✗	✓
	Approach						Estimation Algorithm	Application								
EG-DFA	Ambient	✓	✗	✓	✗	✓	Backlog	Critical/	✓	✗	✗	✗	✗	✓	✗	✗
	Energy Sources						Estimation Algorithm	Urgent Application								
HE-MAC	Generic	✗	✗	✓	✗	✓	N/A	Generic	✓	✗	✗	✗	✓	✗	✗	✗
	Approach							Application								
FSA	Solar Panel	✓	✓	✓	✗	✓	EWMA/	Automatic Meter	✗	✗	✗	✗	✗	✗	✗	✓
							Wake-up/Sleep	Reading Application								
DFSA	Ambient	✓	✗	✓	✓	✓	Markov Chain Model	M2M	✓	✗	✗	✗	✗	✗	✗	✓
	Energy Sources							Application								
EH-RDFSA	Generic	✓	✗	✓	✗	✓	Markov Chain Model	M2M	✓	✗	✗	✗	✓	✗	✓	✗
	Approaches							Application								
AT-MAC	Generic	✗	✓	✓	✗	✓	N/A	Healthcare	✓	N/A	N/A	N/A	✓	✗	✗	✓
	Approach							Monitoring								
PLoRa	Solar Cell	✓	✗	✗	✗	✓	ON-OFF	Various IoT	✓	✗	N/A	N/A	✗	N/A	✗	✓
	and RF Signals						Keying Technique	Application								
SAN	In-Band	✓	✗	✗	✗	✓	SIC/JD	NGIoT	✓	✗	✗	✗	✓	✓	✗	✓
	RF							Application								
Y.Liu	Ambient	✓	✗	✓	✓	✓	N/A	M2M	✓	✗	✗	✗	✗	✗	✗	✗
et al.	Energy Sources							Application								
FarMAC	In-Bnad	✓	✗	✓	✓	✓	Wake-up/Sleep	Data Collection	✓	✗	✗	✗	✗	✓	✗	✓
	RF							Application								
AH-MAC	Solar Panel	✓	✗	✓	✗	✓	Wake-up/Sleep	Low-Rate Monitoring	✓	✓	✓	✗	✓	✓	✗	✓
							Modified	Application/Event-								
							Synchronization	Driven Alarm								
HEH-BMAC	Human Body	✓	✗	✓	✓	✓	Probabilistic Contention	Healthcare	✓	✗	✗	N/A	✓	✗	✗	✗
	Energy Sources							Application								
RIH-MAC	Piezoelectric	✗	✗	✓	✗	✓	RTR Packet/	Nano-Scale	✓	✗	✗	✗	✗	✗	✗	✓
							Coordinated Energy	Monitoring								
							Consumption Schedule									
H-MAC	Solar Cell	✓	✗	✓	✓	✓	Wake-up/Sleep	Heterogeneous	✓	✗	✗	✗	✓	✗	✗	✗
								Networks								
DSR-MAC	Solar Panel	✗	✗	✓	✗	✓	Wake-up/ Sleep	Generic	✓	N/A	N/A	✓	✗	✗	N/A	✗
DSP-MAC							Scheduling	Application								
OD-MAC	Ambient	✗	✓	✓	✗	✓	Opportunistic	Low-Delay/	✓	✗	✓	✗	✗	✓	N/A	✓
	Energy						Forwarding	Delay Sensitive	
	Sources							Application								
A-MAC	Ambient	✗	✓	✓	✗	✓	Opportunistic	Delay-Sensitive	✓	✗	✓	✗	✗	✗	N/A	✗
	Energy						Forwarding	Application	
	Sources															
ERI-MAC	Ambient	✓	✓	✓	✓	✗	Queuing	Realistic	✓	✓	✗	✓	✓	✗	N/A	✓
	Energy						Mechanism	Traffic Model	
	Sources															
RF-AASP	In-Band	✓	✗	✓	✓	✓	Adaptive CW/Adaptive	Application with	✓	✗	✗	✓	✓	✗	N/A	✓
	RF						Beacon Order	Variable Traffic	
							Superframe Order	Conditions								
EA-MAC	In-Band	✓	✗	✓	✗	✓	Adaptive	Environmental	✓	✗	✗	✗	✗	✓	✗	✗
	RF						Contention	Monitoring					
							Algorithm									
SEHEE	Solar Cell	✓	✗	✓	✗	✓	Slotted Preamble	Habitat	✓	✗	✗	✗	✗	✗	N/A	✗
MAC							Technique	Monitoring								
							for Wake-up/	
							Sleep Scheduling									
PS-EHWSN	Generic	✓	✗	✓	✓	✓	Determining Next	N/A	✓	✗	✗	✗	N/A	✗	N/A	✗
MAC	Approach						Period Sleep									
							Period/ LPL									
EEM-EHWSN	Generic	✓	✓	✓	✗	✓	Wake-up/ Sleep	Application with	✓	✗	✓	✗	N/A	✗	✗	✗
MAC	Approach						Scheduling	Periodic Traffic								
WURTICER	Generic	✓	✓	✓	✗	✗	Wake-up/Sleep	Monitoring	✗	✗	✗	✓	N/A	✗	N/A	✗
MAC	Approach						Scheduling	Application								
LEB-MAC	Solar Cell	✓	✗	✓	✓	✓	Fuzzy Logic	N/A	✓	✗	✓	✓	✓	✓	✓	✗
																
ED-MAC	Generic	✓	✗	✓	✗	✓	ED-CR/	N/A	✓	✗	✗	✗	✗	✗	N/A	✗
	Approach						ED-PIR									
SyWiM	Solar Cell	✓	✓	✓	✗	✓	Wake-up Variation	Monitoring	N/A	✓	✓	✓	✓	✓	N/A	✓
							Reduction Power	Application		
							Management									

**Table 10:** Comparison of cross-layer energy harvesting MAC protocols.

Protocol	EH Technique	Energy Efficient	ENO Consideration	Adaption Respect to Energy	Prioritization Respect to Energy	Probabilistic Approach	Energy Technique Management	Application Support	Optimal Path Selection	Adjusted Transmission Power	Number of Connected Layers	Node Grouping
Castagnetti	Solar Cell	✓	✓	✓	✗	✗	Wake-up/Sleep	Monitoring	N/A	✓	2	✗
et al.								Application				
OPWUM	Solar Panel	✓	✗	✓	✗	✓	Wake-up/Sleep/	Monitoring	✓	✗	2	✗
							Timer-based Contention	Application				
TPGFPlus	Solar Cell	✗	✗	✗	✗	✗	Wake-up/Sleep	Generic	✓	✓	3	✓
								Application				
CL_EHSN	In-Band	✓	✓	✓	✗	✓	Wake-up/Sleep	Generic	✓	✓	3	✓
	RF						/Harvesting Time	Application				
LESOP-MAC	N/A	✓	N/A	✓	✗	✓	Wake-up/Sleep	Surveillance	N/A	✗	2	✗
								Application				

## Data Availability

Not applicable for this study.
